# Supplementary material for: Bayesian-frequentist hybrid inference framework for single cell RNA-seq analyses
Source: Hum Genomics. 2024 Jun 20;18:69. doi: 10.1186/s40246-024-00638-0 (PMC11575015; doi:10.1186/s40246-024-00638-0)
Supplement: Supplementary file 1 — Additional file 1: Table S1: The estimation, standard error, 95% confidence interval (95% CI), p-value, adjusted p-value of β1 from the gene detection difference between Hybrid and Bayesian method with informative priors; Table S2: The estimation, standard error, 95% confidence interval (95% CI), p-value, adjusted p-value of β1 from the detailed list of genes detected by Hybrid, non-informative method (43 genes); Table S3: The estimation, standard error, 95% confidence interval (95% CI), p-value, adjusted p-value of β1 from the detailed list of genes detected by Bayesian, informative method (416 genes); Table S4: The estimation, standard error, 95% confidence interval (95% CI), p-value, adjusted p-value of β1 from the detailed list of genes detected by Hybrid, informative method (436 genes); Table S5: A detailed list of pathways detected by Bayesian, informative method (36 pathways); Table S6: A detailed list of pathways detected by Hybrid, informative method (38 pathways). [file 40246_2024_638_MOESM1_ESM.docx]

Supplemental material

**Table S1.** The estimation, standard error, 95% confidence interval (95% CI), p-value, adjusted p-value of $\beta_{1}$ from the gene detection difference between Hybrid and Bayesian method with informative priors.

| **Detection difference** | | | | | | | | | | |
| --- | --- | --- | --- | --- | --- | --- | --- | --- | --- | --- |
|  | **Bayesian, informative** | | | | | **Hybrid, informative** | | | | |
| gene | Estimate | SE | CI | p_value | p_adjust | Estimate | SE | CI | p_value | p_adjust |
| ALOX5 | -0.893 | 0.29 | (-1.462,-0.324) | 0.002 | 0.028 | -0.894 | 0.237 | (-1.358,-0.43) | 0 | 0.002 |
| BPIFB1 | 2.453 | 0.967 | (0.558,4.348) | 0.011 | 0.143 | 2.454 | 0.727 | (1.029,3.878) | 0.001 | 0.01 |
| C1orf162 | -1.321 | 0.455 | (-2.213,-0.43) | 0.004 | 0.049 | -1.323 | 0.387 | (-2.083,-0.564) | 0.001 | 0.009 |
| CARD16 | -0.649 | 0.192 | (-1.026,-0.273) | 0.001 | 0.01 | -0.65 | 0.172 | (-0.986,-0.314) | 0 | 0.002 |
| CCL24 | 1.512 | 0.453 | (0.623,2.4) | 0.001 | 0.012 | 1.511 | 0.349 | (0.827,2.196) | 0 | 0 |
| CSTA | -0.748 | 0.239 | (-1.217,-0.279) | 0.002 | 0.024 | -0.749 | 0.191 | (-1.124,-0.374) | 0 | 0.001 |
| CTSK | 0.706 | 0.213 | (0.288,1.125) | 0.001 | 0.013 | 0.706 | 0.173 | (0.368,1.045) | 0 | 0.001 |
| FPR1 | -0.59 | 0.192 | (-0.966,-0.213) | 0.002 | 0.029 | -0.59 | 0.155 | (-0.894,-0.287) | 0 | 0.002 |
| GCA | -0.657 | 0.195 | (-1.039,-0.275) | 0.001 | 0.01 | -0.658 | 0.163 | (-0.978,-0.338) | 0 | 0.001 |
| GYPC | -0.683 | 0.206 | (-1.087,-0.279) | 0.001 | 0.013 | -0.684 | 0.174 | (-1.025,-0.343) | 0 | 0.001 |
| IFI27 | -0.851 | 0.279 | (-1.397,-0.304) | 0.002 | 0.031 | -0.852 | 0.243 | (-1.328,-0.375) | 0 | 0.006 |
| MT1X | 0.924 | 0.343 | (0.251,1.597) | 0.007 | 0.093 | 0.923 | 0.269 | (0.396,1.45) | 0.001 | 0.008 |
| NCF2 | -0.787 | 0.276 | (-1.328,-0.247) | 0.004 | 0.057 | -0.788 | 0.228 | (-1.235,-0.342) | 0.001 | 0.007 |
| PDK4 | -1.288 | 0.473 | (-2.216,-0.361) | 0.006 | 0.085 | -1.29 | 0.367 | (-2.01,-0.571) | 0 | 0.006 |
| RGS1 | 1.214 | 0.359 | (0.51,1.918) | 0.001 | 0.01 | 1.213 | 0.277 | (0.671,1.755) | 0 | 0 |
| RHOG | -0.659 | 0.196 | (-1.044,-0.275) | 0.001 | 0.011 | -0.66 | 0.16 | (-0.974,-0.346) | 0 | 0.001 |
| SLC7A7 | -0.994 | 0.305 | (-1.593,-0.396) | 0.001 | 0.016 | -0.995 | 0.261 | (-1.507,-0.482) | 0 | 0.002 |
| SNX10 | -1.02 | 0.359 | (-1.724,-0.316) | 0.005 | 0.06 | -1.021 | 0.293 | (-1.596,-0.447) | 0 | 0.007 |
| TREM1 | -1.121 | 0.345 | (-1.797,-0.446) | 0.001 | 0.016 | -1.123 | 0.279 | (-1.67,-0.577) | 0 | 0.001 |
| VSIG4 | -2.128 | 0.7 | (-3.501,-0.756) | 0.002 | 0.032 | -2.132 | 0.591 | (-3.289,-0.974) | 0 | 0.004 |

**Table S2.** The estimation, standard error, 95% confidence interval (95% CI), p-value, adjusted p-value of $\beta_{1}$ from the detailed list of genes detected by Hybrid, non-informative method (43 genes).

| **Hybrid, non-informative** | | | | | |
| --- | --- | --- | --- | --- | --- |
| gene | Estimate | SE | CI | p_value | p_adjust |
| C1QC | -17.356 | 3.255 | (-23.736,-10.976) | 0 | 0 |
| TXNIP | -5.73 | 1.046 | (-7.779,-3.68) | 0 | 0 |
| CD302 | -1.034 | 0.132 | (-1.292,-0.776) | 0 | 0 |
| FN1 | 5.829 | 0.962 | (3.944,7.713) | 0 | 0 |
| PLA2G7 | 0.916 | 0.177 | (0.569,1.263) | 0 | 0 |
| CITED2 | -2.367 | 0.46 | (-3.27,-1.465) | 0 | 0 |
| EZR | 0.896 | 0.142 | (0.618,1.174) | 0 | 0 |
| VSIG4 | -3.653 | 0.696 | (-5.018,-2.288) | 0 | 0 |
| EMP1 | 1.462 | 0.27 | (0.932,1.991) | 0 | 0 |
| LGMN | 1.637 | 0.282 | (1.084,2.19) | 0 | 0 |
| C15orf48 | 2.506 | 0.363 | (1.794,3.218) | 0 | 0 |
| GLDN | -1.388 | 0.201 | (-1.782,-0.994) | 0 | 0 |
| MMP9 | 1.356 | 0.269 | (0.83,1.882) | 0 | 0 |
| EMP3 | 3.181 | 0.541 | (2.12,4.242) | 0 | 0 |
| LILRB4 | 0.687 | 0.117 | (0.458,0.916) | 0 | 0 |
| CTSK | 0.998 | 0.204 | (0.598,1.399) | 0 | 0.001 |
| TKT | -2.558 | 0.523 | (-3.583,-1.532) | 0 | 0.001 |
| CD163 | -4.581 | 0.955 | (-6.453,-2.708) | 0 | 0.001 |
| CLEC7A | -0.8 | 0.167 | (-1.128,-0.471) | 0 | 0.001 |
| CRIP1 | 7.585 | 1.615 | (4.419,10.751) | 0 | 0.001 |
| RGS1 | 1.442 | 0.316 | (0.824,2.061) | 0 | 0.002 |
| AKR1C3 | -1.311 | 0.292 | (-1.883,-0.739) | 0 | 0.002 |
| MT1X | 1.386 | 0.3 | (0.798,1.974) | 0 | 0.002 |
| GPX3 | -0.745 | 0.167 | (-1.073,-0.417) | 0 | 0.003 |
| C1QB | -28.115 | 6.525 | (-40.904,-15.326) | 0 | 0.004 |
| CHIT1 | 1.529 | 0.352 | (0.839,2.219) | 0 | 0.004 |
| SLC7A7 | -1.501 | 0.345 | (-2.177,-0.825) | 0 | 0.004 |
| PGD | -2.041 | 0.478 | (-2.979,-1.104) | 0 | 0.005 |
| C1QA | -20.667 | 4.86 | (-30.193,-11.141) | 0 | 0.005 |
| C1orf162 | -2.133 | 0.496 | (-3.105,-1.162) | 0 | 0.005 |
| CRTAP | -0.894 | 0.209 | (-1.304,-0.485) | 0 | 0.005 |
| TBXAS1 | -0.851 | 0.199 | (-1.24,-0.461) | 0 | 0.005 |
| GLUL | -7.85 | 1.889 | (-11.552,-4.147) | 0 | 0.007 |
| MCOLN1 | -0.593 | 0.142 | (-0.872,-0.315) | 0 | 0.007 |
| CIRBP | -1.059 | 0.256 | (-1.56,-0.558) | 0 | 0.008 |
| S100A10 | 13.53 | 3.306 | (7.05,20.011) | 0 | 0.009 |
| PDK4 | -1.716 | 0.418 | (-2.535,-0.896) | 0 | 0.009 |
| EIF3F | -1.166 | 0.285 | (-1.725,-0.607) | 0 | 0.009 |
| AKR1C2 | -0.77 | 0.188 | (-1.139,-0.4) | 0 | 0.009 |
| RGCC | 2.52 | 0.614 | (1.317,3.723) | 0 | 0.009 |
| CCL2 | 6.905 | 1.687 | (3.599,10.211) | 0 | 0.009 |
| HCLS1 | -0.997 | 0.245 | (-1.477,-0.517) | 0 | 0.01 |
| EIF3H | -1.058 | 0.261 | (-1.569,-0.547) | 0 | 0.01 |

**Table S3.** The estimation, standard error, 95% confidence interval (95% CI), p-value, adjusted p-value of $\beta_{1}$ from the detailed list of genes detected by Bayesian, informative method (416 genes).

| **Bayesian, informative** | | | | | |
| --- | --- | --- | --- | --- | --- |
| gene | Estimate | SE | CI | p_value | p_adjust |
| PRKCZ | -0.601 | 0.006 | (-0.612,-0.59) | 0 | 0 |
| TP73 | 0.659 | 0.002 | (0.656,0.662) | 0 | 0 |
| PLA2G2A | 1.597 | 0.297 | (1.014,2.179) | 0 | 0 |
| FCN3 | -1.29 | 0.019 | (-1.328,-1.252) | 0 | 0 |
| CD164L2 | 0.672 | 0.005 | (0.662,0.683) | 0 | 0 |
| CSF3R | -0.893 | 0.075 | (-1.04,-0.745) | 0 | 0 |
| MACF1 | -0.619 | 0.049 | (-0.716,-0.522) | 0 | 0 |
| FAM183A | 0.673 | 0.029 | (0.615,0.731) | 0 | 0 |
| CCDC17 | 0.586 | 0.005 | (0.577,0.596) | 0 | 0 |
| TSPAN1 | 0.861 | 0.041 | (0.78,0.941) | 0 | 0 |
| ERICH3 | 0.635 | 0.005 | (0.626,0.645) | 0 | 0 |
| GBP4 | -0.817 | 0.032 | (-0.88,-0.754) | 0 | 0 |
| S1PR1 | -0.7 | 0.003 | (-0.707,-0.694) | 0 | 0 |
| C1orf194 | 0.737 | 0.031 | (0.675,0.799) | 0 | 0 |
| PIFO | 0.654 | 0.022 | (0.612,0.697) | 0 | 0 |
| SPAG17 | 0.741 | 0.004 | (0.733,0.748) | 0 | 0 |
| FMO5 | -0.711 | 0.01 | (-0.731,-0.69) | 0 | 0 |
| S100A2 | 1.842 | 0.206 | (1.439,2.245) | 0 | 0 |
| SLC27A3 | -0.598 | 0.049 | (-0.693,-0.502) | 0 | 0 |
| KCNN3 | 1.034 | 0.001 | (1.032,1.037) | 0 | 0 |
| CRABP2 | 0.712 | 0.032 | (0.649,0.774) | 0 | 0 |
| ACKR1 | 1.018 | 0.043 | (0.934,1.102) | 0 | 0 |
| CFAP126 | 0.703 | 0.008 | (0.687,0.718) | 0 | 0 |
| RCSD1 | -0.861 | 0.043 | (-0.945,-0.777) | 0 | 0 |
| KIAA0040 | -0.689 | 0.011 | (-0.711,-0.668) | 0 | 0 |
| APOBEC4 | 0.704 | 0.002 | (0.7,0.708) | 0 | 0 |
| CFH | 0.818 | 0.03 | (0.758,0.877) | 0 | 0 |
| CTSE | 0.696 | 0.015 | (0.667,0.725) | 0 | 0 |
| PPP2R5A | -0.591 | 0.018 | (-0.626,-0.556) | 0 | 0 |
| C1orf198 | -0.859 | 0.004 | (-0.867,-0.851) | 0 | 0 |
| FAM89A | -0.762 | 0.118 | (-0.993,-0.531) | 0 | 0 |
| OPN3 | -0.647 | 0.103 | (-0.849,-0.444) | 0 | 0 |
| KLF11 | -0.655 | 0.02 | (-0.693,-0.617) | 0 | 0 |
| HPCAL1 | -1.095 | 0.094 | (-1.279,-0.911) | 0 | 0 |
| DRC1 | 0.59 | 0.003 | (0.584,0.595) | 0 | 0 |
| PRKCE | -0.754 | 0.024 | (-0.802,-0.707) | 0 | 0 |
| EPAS1 | -0.774 | 0.051 | (-0.874,-0.674) | 0 | 0 |
| SPTBN1 | -0.65 | 0.018 | (-0.685,-0.614) | 0 | 0 |
| GNLY | -0.828 | 0.133 | (-1.088,-0.568) | 0 | 0 |
| VWA3B | 0.61 | 0.003 | (0.605,0.616) | 0 | 0 |
| IL1RL1 | -0.819 | 0.018 | (-0.854,-0.785) | 0 | 0 |
| FHL2 | 1.24 | 0.023 | (1.195,1.286) | 0 | 0 |
| EPB41L5 | -1.094 | 0.003 | (-1.1,-1.088) | 0 | 0 |
| LIMS2 | -0.71 | 0.019 | (-0.747,-0.673) | 0 | 0 |
| CCDC74B | 0.586 | 0.003 | (0.58,0.591) | 0 | 0 |
| CCDC74A | 0.586 | 0.005 | (0.576,0.596) | 0 | 0 |
| MAP3K19 | 0.646 | 0.002 | (0.642,0.65) | 0 | 0 |
| CD302 | -1.22 | 0.128 | (-1.471,-0.969) | 0 | 0 |
| ZNF385B | -0.958 | 0.008 | (-0.973,-0.943) | 0 | 0 |
| CALCRL | -0.61 | 0.013 | (-0.636,-0.583) | 0 | 0 |
| TFPI | -0.649 | 0.059 | (-0.764,-0.534) | 0 | 0 |
| COL3A1 | 1.17 | 0.124 | (0.926,1.414) | 0 | 0 |
| SLC40A1 | -0.622 | 0.031 | (-0.684,-0.561) | 0 | 0 |
| ANKRD44 | -0.608 | 0.04 | (-0.686,-0.53) | 0 | 0 |
| KIAA2012 | 0.627 | 0.001 | (0.625,0.63) | 0 | 0 |
| BMPR2 | -0.615 | 0.017 | (-0.647,-0.582) | 0 | 0 |
| MDH1B | 0.602 | 0.002 | (0.599,0.606) | 0 | 0 |
| IGFBP2 | 0.884 | 0.139 | (0.611,1.157) | 0 | 0 |
| TNS1 | -0.623 | 0.02 | (-0.662,-0.583) | 0 | 0 |
| CFAP65 | 0.635 | 0.003 | (0.629,0.64) | 0 | 0 |
| TUBA4B | 0.73 | 0.005 | (0.721,0.739) | 0 | 0 |
| COL6A3 | 0.804 | 0.033 | (0.74,0.868) | 0 | 0 |
| RAMP1 | 0.618 | 0.009 | (0.601,0.636) | 0 | 0 |
| PPARG | -1.268 | 0.287 | (-1.831,-0.706) | 0 | 0 |
| FBLN2 | 0.86 | 0.021 | (0.819,0.901) | 0 | 0 |
| SH3BP5 | -0.653 | 0.114 | (-0.877,-0.429) | 0 | 0 |
| GPD1L | -0.614 | 0.003 | (-0.62,-0.609) | 0 | 0 |
| STAC | -0.611 | 0.084 | (-0.777,-0.445) | 0 | 0 |
| VIPR1 | -1.195 | 0.004 | (-1.203,-1.187) | 0 | 0 |
| ABHD5 | -0.972 | 0.202 | (-1.369,-0.576) | 0 | 0 |
| CCRL2 | -0.631 | 0.055 | (-0.739,-0.523) | 0 | 0 |
| CDHR4 | 0.696 | 0.003 | (0.69,0.701) | 0 | 0 |
| HYAL2 | -0.656 | 0.014 | (-0.684,-0.629) | 0 | 0 |
| ZMYND10 | 0.594 | 0.012 | (0.569,0.618) | 0 | 0 |
| CACNA2D2 | -0.91 | 0.005 | (-0.92,-0.9) | 0 | 0 |
| TNNC1 | -1.086 | 0.006 | (-1.097,-1.076) | 0 | 0 |
| DNAH12 | 0.671 | 0.009 | (0.654,0.689) | 0 | 0 |
| FAM107A | -0.738 | 0.004 | (-0.745,-0.732) | 0 | 0 |
| SNTN | 0.739 | 0.025 | (0.689,0.788) | 0 | 0 |
| TMEM45A | 0.869 | 0.02 | (0.83,0.908) | 0 | 0 |
| HHLA2 | 1.104 | 0.001 | (1.101,1.106) | 0 | 0 |
| CCDC80 | 0.957 | 0.148 | (0.666,1.247) | 0 | 0 |
| ARHGAP31 | -0.687 | 0.026 | (-0.737,-0.637) | 0 | 0 |
| CFAP100 | 0.624 | 0.003 | (0.618,0.629) | 0 | 0 |
| AMOTL2 | -0.643 | 0.003 | (-0.648,-0.638) | 0 | 0 |
| CLDN18 | -0.788 | 0.016 | (-0.819,-0.756) | 0 | 0 |
| PCOLCE2 | -0.627 | 0.13 | (-0.881,-0.373) | 0 | 0 |
| CP | 1 | 0.025 | (0.952,1.048) | 0 | 0 |
| ANKUB1 | 0.625 | 0.002 | (0.622,0.629) | 0 | 0 |
| MME | -1.221 | 0.087 | (-1.391,-1.051) | 0 | 0 |
| VEPH1 | -0.596 | 0.01 | (-0.616,-0.576) | 0 | 0 |
| RARRES1 | 1.018 | 0.113 | (0.797,1.239) | 0 | 0 |
| ZBBX | 0.702 | 0.003 | (0.696,0.708) | 0 | 0 |
| LAMP3 | -0.765 | 0.03 | (-0.824,-0.706) | 0 | 0 |
| ST6GAL1 | 0.649 | 0.012 | (0.627,0.672) | 0 | 0 |
| P3H2 | -0.871 | 0.01 | (-0.891,-0.851) | 0 | 0 |
| CLDN1 | 0.817 | 0.046 | (0.728,0.907) | 0 | 0 |
| MUC4 | 1.008 | 0.011 | (0.986,1.03) | 0 | 0 |
| CD38 | 0.746 | 0.012 | (0.722,0.769) | 0 | 0 |
| PROM1 | 0.954 | 0.006 | (0.942,0.966) | 0 | 0 |
| LIMCH1 | -0.816 | 0.006 | (-0.826,-0.805) | 0 | 0 |
| ATP8A1 | -0.703 | 0.003 | (-0.709,-0.696) | 0 | 0 |
| HOPX | -0.672 | 0.152 | (-0.971,-0.373) | 0 | 0 |
| CXCL6 | 0.766 | 0.009 | (0.749,0.784) | 0 | 0 |
| CDKL2 | -0.622 | 0.004 | (-0.63,-0.614) | 0 | 0 |
| ANXA3 | -0.993 | 0.009 | (-1.01,-0.975) | 0 | 0 |
| ADH1B | -0.682 | 0.094 | (-0.867,-0.497) | 0 | 0 |
| SLC39A8 | -0.945 | 0.036 | (-1.016,-0.874) | 0 | 0 |
| NPNT | -0.725 | 0.003 | (-0.73,-0.719) | 0 | 0 |
| CFI | 0.647 | 0.009 | (0.63,0.663) | 0 | 0 |
| HHIP | -0.81 | 0.009 | (-0.828,-0.792) | 0 | 0 |
| TTC29 | 0.602 | 0.003 | (0.596,0.608) | 0 | 0 |
| MSMO1 | -0.603 | 0.03 | (-0.661,-0.546) | 0 | 0 |
| HPGD | -1.173 | 0.221 | (-1.605,-0.741) | 0 | 0 |
| SPATA4 | 0.589 | 0.001 | (0.586,0.592) | 0 | 0 |
| WWC2 | -1.01 | 0.008 | (-1.026,-0.994) | 0 | 0 |
| C5orf38 | -0.726 | 0.018 | (-0.762,-0.69) | 0 | 0 |
| C5orf49 | 0.66 | 0.015 | (0.629,0.69) | 0 | 0 |
| CAPSL | 0.753 | 0.014 | (0.727,0.78) | 0 | 0 |
| LIFR | -0.904 | 0.003 | (-0.91,-0.898) | 0 | 0 |
| C6 | 0.753 | 0.002 | (0.749,0.757) | 0 | 0 |
| HMGCS1 | -0.627 | 0.016 | (-0.658,-0.596) | 0 | 0 |
| CCNO | 0.904 | 0.009 | (0.885,0.922) | 0 | 0 |
| OCLN | -0.766 | 0.005 | (-0.775,-0.757) | 0 | 0 |
| FAM81B | 0.697 | 0.007 | (0.683,0.711) | 0 | 0 |
| PDLIM4 | 1.213 | 0.022 | (1.17,1.255) | 0 | 0 |
| CXCL14 | 1.381 | 0.017 | (1.349,1.414) | 0 | 0 |
| MZB1 | 1.377 | 0.136 | (1.11,1.644) | 0 | 0 |
| ADRB2 | -0.92 | 0.02 | (-0.959,-0.881) | 0 | 0 |
| GPX3 | -1.172 | 0.165 | (-1.494,-0.849) | 0 | 0 |
| PHACTR1 | -0.738 | 0.173 | (-1.077,-0.4) | 0 | 0 |
| MYLIP | -0.615 | 0.025 | (-0.664,-0.567) | 0 | 0 |
| STMND1 | 0.59 | 0.003 | (0.584,0.596) | 0 | 0 |
| RNF144B | -0.871 | 0.099 | (-1.065,-0.677) | 0 | 0 |
| SFTA2 | -0.83 | 0.141 | (-1.105,-0.554) | 0 | 0 |
| SLC44A4 | 0.63 | 0.013 | (0.603,0.656) | 0 | 0 |
| CFB | 0.775 | 0.015 | (0.745,0.805) | 0 | 0 |
| AGER | -1.459 | 0.009 | (-1.476,-1.442) | 0 | 0 |
| CCND3 | -0.628 | 0.079 | (-0.782,-0.474) | 0 | 0 |
| CLIC5 | -0.666 | 0.002 | (-0.67,-0.662) | 0 | 0 |
| PLA2G7 | 0.938 | 0.188 | (0.569,1.307) | 0 | 0 |
| ANKRD66 | 0.66 | 0.004 | (0.652,0.668) | 0 | 0 |
| ADGRF5 | -0.735 | 0.01 | (-0.754,-0.716) | 0 | 0 |
| GSTA1 | 0.993 | 0.041 | (0.913,1.074) | 0 | 0 |
| TPBG | 0.767 | 0.006 | (0.756,0.778) | 0 | 0 |
| CD24 | 0.683 | 0.036 | (0.613,0.753) | 0 | 0 |
| RSPH4A | 0.594 | 0.003 | (0.588,0.6) | 0 | 0 |
| ECT2L | 0.625 | 0.002 | (0.621,0.628) | 0 | 0 |
| PHACTR2 | -0.624 | 0.025 | (-0.672,-0.576) | 0 | 0 |
| STX11 | -1.06 | 0.086 | (-1.229,-0.89) | 0 | 0 |
| QKI | -0.667 | 0.053 | (-0.771,-0.563) | 0 | 0 |
| C6orf118 | 0.7 | 0.003 | (0.694,0.707) | 0 | 0 |
| ETV1 | -0.614 | 0.005 | (-0.624,-0.605) | 0 | 0 |
| RAPGEF5 | -0.788 | 0.002 | (-0.791,-0.784) | 0 | 0 |
| RAMP3 | -0.749 | 0.005 | (-0.759,-0.74) | 0 | 0 |
| ABCA13 | 0.675 | 0.002 | (0.671,0.679) | 0 | 0 |
| HIP1 | -0.632 | 0.017 | (-0.664,-0.599) | 0 | 0 |
| UPK3B | -0.765 | 0.01 | (-0.785,-0.744) | 0 | 0 |
| CD36 | -0.789 | 0.122 | (-1.028,-0.549) | 0 | 0 |
| PILRA | -0.718 | 0.136 | (-0.984,-0.452) | 0 | 0 |
| MUC12 | 0.623 | 0.01 | (0.603,0.643) | 0 | 0 |
| EFCAB10 | 0.607 | 0.004 | (0.6,0.614) | 0 | 0 |
| DOCK4 | -0.634 | 0.029 | (-0.691,-0.577) | 0 | 0 |
| CAV2 | -0.591 | 0.009 | (-0.61,-0.573) | 0 | 0 |
| CAV1 | -0.935 | 0.027 | (-0.988,-0.882) | 0 | 0 |
| TSPAN12 | -0.873 | 0.005 | (-0.882,-0.864) | 0 | 0 |
| WWC3 | -0.602 | 0.004 | (-0.61,-0.593) | 0 | 0 |
| ARHGAP6 | -0.828 | 0.017 | (-0.863,-0.794) | 0 | 0 |
| TSPAN7 | -0.597 | 0.005 | (-0.607,-0.586) | 0 | 0 |
| MID1IP1 | -0.74 | 0.049 | (-0.836,-0.644) | 0 | 0 |
| MAOA | -0.745 | 0.016 | (-0.777,-0.713) | 0 | 0 |
| EFHC2 | 0.644 | 0.002 | (0.641,0.647) | 0 | 0 |
| PIM2 | 0.813 | 0.017 | (0.778,0.847) | 0 | 0 |
| MSN | -0.872 | 0.144 | (-1.154,-0.591) | 0 | 0 |
| PIH1D3 | 0.674 | 0.003 | (0.668,0.68) | 0 | 0 |
| AKAP14 | 0.647 | 0.005 | (0.637,0.656) | 0 | 0 |
| GPC3 | -0.634 | 0.004 | (-0.642,-0.627) | 0 | 0 |
| HMGB3 | 0.625 | 0.021 | (0.584,0.665) | 0 | 0 |
| DLC1 | -0.708 | 0.014 | (-0.735,-0.68) | 0 | 0 |
| DOK2 | -0.819 | 0.165 | (-1.143,-0.495) | 0 | 0 |
| PEBP4 | -0.879 | 0.018 | (-0.914,-0.843) | 0 | 0 |
| ADAM28 | 0.695 | 0.013 | (0.669,0.72) | 0 | 0 |
| BNIP3L | -0.686 | 0.158 | (-0.995,-0.376) | 0 | 0 |
| RAB11FIP1 | -0.691 | 0.091 | (-0.87,-0.513) | 0 | 0 |
| EFCAB1 | 0.629 | 0.011 | (0.607,0.65) | 0 | 0 |
| PPP1R42 | 0.632 | 0.002 | (0.628,0.636) | 0 | 0 |
| HEY1 | -0.741 | 0.005 | (-0.752,-0.731) | 0 | 0 |
| PAG1 | -0.765 | 0.089 | (-0.939,-0.59) | 0 | 0 |
| CA2 | -0.824 | 0.093 | (-1.007,-0.642) | 0 | 0 |
| GEM | 0.755 | 0.137 | (0.488,1.023) | 0 | 0 |
| COL14A1 | 1.311 | 0.031 | (1.25,1.371) | 0 | 0 |
| FBXO32 | 0.611 | 0.006 | (0.599,0.623) | 0 | 0 |
| DENND3 | -0.807 | 0.019 | (-0.844,-0.77) | 0 | 0 |
| LY6D | 1.368 | 0.013 | (1.344,1.393) | 0 | 0 |
| CDKN2A | 0.64 | 0.011 | (0.619,0.66) | 0 | 0 |
| C9orf24 | 0.729 | 0.037 | (0.656,0.802) | 0 | 0 |
| DNAI1 | 0.674 | 0.002 | (0.669,0.678) | 0 | 0 |
| UNC13B | -0.702 | 0.006 | (-0.715,-0.689) | 0 | 0 |
| PIP5K1B | -0.835 | 0.004 | (-0.842,-0.828) | 0 | 0 |
| C9orf135 | 0.747 | 0.006 | (0.735,0.76) | 0 | 0 |
| PRUNE2 | 0.599 | 0.006 | (0.588,0.61) | 0 | 0 |
| AGTPBP1 | -0.759 | 0.027 | (-0.812,-0.706) | 0 | 0 |
| GOLM1 | 0.765 | 0.007 | (0.751,0.778) | 0 | 0 |
| TNC | 0.788 | 0.008 | (0.772,0.803) | 0 | 0 |
| MORN5 | 0.627 | 0.009 | (0.609,0.645) | 0 | 0 |
| WDR38 | 0.703 | 0.009 | (0.685,0.721) | 0 | 0 |
| SCAI | -0.659 | 0.005 | (-0.668,-0.65) | 0 | 0 |
| CFAP157 | 0.623 | 0.008 | (0.608,0.638) | 0 | 0 |
| CFAP77 | 0.696 | 0.003 | (0.69,0.702) | 0 | 0 |
| EGFL7 | -0.697 | 0.014 | (-0.724,-0.67) | 0 | 0 |
| CLIC3 | -1.012 | 0.025 | (-1.061,-0.964) | 0 | 0 |
| LRRC26 | 0.801 | 0.008 | (0.786,0.816) | 0 | 0 |
| MUC5B | 1.258 | 0.093 | (1.076,1.44) | 0 | 0 |
| SYT8 | 0.8 | 0.015 | (0.771,0.83) | 0 | 0 |
| C11orf16 | 0.635 | 0.002 | (0.631,0.639) | 0 | 0 |
| SAA2 | 0.704 | 0.039 | (0.626,0.781) | 0 | 0 |
| MDK | 0.656 | 0.041 | (0.575,0.737) | 0 | 0 |
| MS4A8 | 0.673 | 0.012 | (0.65,0.696) | 0 | 0 |
| ASRGL1 | -0.653 | 0.02 | (-0.692,-0.615) | 0 | 0 |
| CTSW | -0.634 | 0.045 | (-0.723,-0.545) | 0 | 0 |
| DNAJB13 | 0.628 | 0.003 | (0.623,0.633) | 0 | 0 |
| ARRB1 | -0.824 | 0.021 | (-0.866,-0.782) | 0 | 0 |
| GAB2 | -0.605 | 0.024 | (-0.651,-0.559) | 0 | 0 |
| C11orf97 | 0.697 | 0.004 | (0.689,0.706) | 0 | 0 |
| C11orf88 | 0.606 | 0.015 | (0.576,0.636) | 0 | 0 |
| FXYD6 | -0.633 | 0.017 | (-0.666,-0.599) | 0 | 0 |
| TMPRSS4 | 1.467 | 0.006 | (1.455,1.479) | 0 | 0 |
| JAML | -0.59 | 0.103 | (-0.791,-0.389) | 0 | 0 |
| THY1 | 1.291 | 0.032 | (1.229,1.353) | 0 | 0 |
| UBASH3B | -0.799 | 0.081 | (-0.958,-0.64) | 0 | 0 |
| NRGN | -0.742 | 0.013 | (-0.768,-0.715) | 0 | 0 |
| VSIG2 | -0.623 | 0.008 | (-0.638,-0.608) | 0 | 0 |
| ESAM | -0.906 | 0.006 | (-0.919,-0.894) | 0 | 0 |
| ECHDC3 | -0.592 | 0.036 | (-0.662,-0.522) | 0 | 0 |
| NEBL | -0.778 | 0.004 | (-0.786,-0.769) | 0 | 0 |
| SPAG6 | 0.69 | 0.007 | (0.677,0.703) | 0 | 0 |
| ARMC3 | 0.692 | 0.004 | (0.685,0.699) | 0 | 0 |
| OTUD1 | -0.721 | 0.047 | (-0.813,-0.628) | 0 | 0 |
| ENKUR | 0.643 | 0.006 | (0.631,0.654) | 0 | 0 |
| CXCL12 | 0.618 | 0.059 | (0.502,0.734) | 0 | 0 |
| MSMB | 1.045 | 0.112 | (0.826,1.264) | 0 | 0 |
| FRMPD2 | 0.769 | 0.001 | (0.767,0.771) | 0 | 0 |
| STOX1 | 0.619 | 0.002 | (0.614,0.623) | 0 | 0 |
| PRF1 | -0.809 | 0.017 | (-0.843,-0.775) | 0 | 0 |
| SPOCK2 | -1.001 | 0.004 | (-1.009,-0.992) | 0 | 0 |
| SFTPD | -0.65 | 0.044 | (-0.736,-0.563) | 0 | 0 |
| DYDC2 | 0.59 | 0.003 | (0.583,0.597) | 0 | 0 |
| ADIRF | -0.801 | 0.059 | (-0.916,-0.686) | 0 | 0 |
| PAPSS2 | -0.844 | 0.158 | (-1.154,-0.533) | 0 | 0 |
| IFIT2 | -0.646 | 0.063 | (-0.77,-0.523) | 0 | 0 |
| IFIT3 | -0.737 | 0.12 | (-0.972,-0.502) | 0 | 0 |
| FFAR4 | -0.741 | 0.05 | (-0.839,-0.643) | 0 | 0 |
| CRTAC1 | -1.09 | 0.008 | (-1.106,-1.075) | 0 | 0 |
| CFAP43 | 0.621 | 0.005 | (0.611,0.632) | 0 | 0 |
| PLEKHS1 | 1.041 | 0.01 | (1.022,1.061) | 0 | 0 |
| PLEKHA1 | -0.589 | 0.008 | (-0.606,-0.573) | 0 | 0 |
| FANK1 | 0.634 | 0.004 | (0.627,0.641) | 0 | 0 |
| CFAP46 | 0.647 | 0.001 | (0.644,0.649) | 0 | 0 |
| NINJ2 | -0.755 | 0.017 | (-0.789,-0.721) | 0 | 0 |
| CCND2 | 0.744 | 0.031 | (0.682,0.805) | 0 | 0 |
| CLEC4E | -0.741 | 0.072 | (-0.881,-0.6) | 0 | 0 |
| KLRB1 | -0.621 | 0.009 | (-0.639,-0.602) | 0 | 0 |
| CLEC12A | -0.78 | 0.094 | (-0.964,-0.596) | 0 | 0 |
| CLEC7A | -0.803 | 0.16 | (-1.117,-0.489) | 0 | 0 |
| GABARAPL1 | -0.646 | 0.156 | (-0.951,-0.341) | 0 | 0 |
| KLRD1 | -0.829 | 0.018 | (-0.865,-0.793) | 0 | 0 |
| PPFIBP1 | -0.684 | 0.009 | (-0.702,-0.666) | 0 | 0 |
| LRRK2 | -0.658 | 0.034 | (-0.725,-0.591) | 0 | 0 |
| AMIGO2 | -0.611 | 0.058 | (-0.724,-0.498) | 0 | 0 |
| FKBP11 | 0.731 | 0.027 | (0.679,0.784) | 0 | 0 |
| AQP5 | 0.991 | 0.035 | (0.923,1.06) | 0 | 0 |
| ACVRL1 | -0.953 | 0.029 | (-1.011,-0.896) | 0 | 0 |
| GRASP | -0.643 | 0.046 | (-0.734,-0.553) | 0 | 0 |
| KRT5 | 1.466 | 0.03 | (1.407,1.525) | 0 | 0 |
| RBMS2 | -0.666 | 0.029 | (-0.722,-0.61) | 0 | 0 |
| HSD17B6 | -1.126 | 0.012 | (-1.15,-1.102) | 0 | 0 |
| PTPRB | -0.936 | 0.002 | (-0.939,-0.932) | 0 | 0 |
| TSPAN19 | 0.636 | 0.007 | (0.622,0.649) | 0 | 0 |
| LRRIQ1 | 0.597 | 0.008 | (0.581,0.613) | 0 | 0 |
| CHPT1 | -0.677 | 0.062 | (-0.798,-0.557) | 0 | 0 |
| IGF1 | 1.088 | 0.058 | (0.974,1.201) | 0 | 0 |
| SELPLG | -0.588 | 0.094 | (-0.773,-0.403) | 0 | 0 |
| TRPV4 | 0.663 | 0.008 | (0.648,0.678) | 0 | 0 |
| CFAP73 | 0.655 | 0.005 | (0.646,0.664) | 0 | 0 |
| CCDC60 | 0.668 | 0.002 | (0.664,0.671) | 0 | 0 |
| PLA2G1B | -0.937 | 0.018 | (-0.973,-0.902) | 0 | 0 |
| OASL | -0.857 | 0.092 | (-1.038,-0.676) | 0 | 0 |
| LRRC43 | 0.616 | 0.001 | (0.613,0.618) | 0 | 0 |
| FRY | -0.752 | 0.007 | (-0.766,-0.738) | 0 | 0 |
| CCNA1 | 0.66 | 0.003 | (0.654,0.665) | 0 | 0 |
| POSTN | 1.317 | 0.019 | (1.279,1.355) | 0 | 0 |
| STOML3 | 0.703 | 0.003 | (0.698,0.709) | 0 | 0 |
| FAM216B | 0.691 | 0.008 | (0.675,0.707) | 0 | 0 |
| SPRYD7 | -0.779 | 0.016 | (-0.809,-0.748) | 0 | 0 |
| LMO7 | -0.708 | 0.012 | (-0.732,-0.685) | 0 | 0 |
| EDNRB | -1.021 | 0.009 | (-1.039,-1.004) | 0 | 0 |
| DOCK9 | -0.63 | 0.002 | (-0.633,-0.627) | 0 | 0 |
| CLEC14A | -0.729 | 0.004 | (-0.737,-0.721) | 0 | 0 |
| SIX1 | 0.906 | 0.004 | (0.899,0.914) | 0 | 0 |
| JDP2 | -0.663 | 0.029 | (-0.72,-0.607) | 0 | 0 |
| TJP1 | -0.665 | 0.004 | (-0.674,-0.657) | 0 | 0 |
| C15orf48 | 1.814 | 0.38 | (1.069,2.559) | 0 | 0 |
| GLDN | -1.242 | 0.209 | (-1.651,-0.833) | 0 | 0 |
| CCDC33 | 0.732 | 0.001 | (0.73,0.734) | 0 | 0 |
| CFAP161 | -0.614 | 0.001 | (-0.617,-0.612) | 0 | 0 |
| ALDH1A3 | 1.143 | 0.038 | (1.069,1.217) | 0 | 0 |
| HBA2 | -0.737 | 0.068 | (-0.869,-0.604) | 0 | 0 |
| CCDC78 | 0.649 | 0.011 | (0.627,0.67) | 0 | 0 |
| SLC9A3R2 | -0.905 | 0.014 | (-0.933,-0.877) | 0 | 0 |
| ABCA3 | -0.675 | 0.011 | (-0.696,-0.653) | 0 | 0 |
| EMP2 | -0.674 | 0.017 | (-0.706,-0.641) | 0 | 0 |
| DNAH3 | 0.624 | 0.002 | (0.62,0.628) | 0 | 0 |
| SULT1A1 | -0.749 | 0.046 | (-0.84,-0.659) | 0 | 0 |
| SPN | -0.692 | 0.09 | (-0.869,-0.515) | 0 | 0 |
| MMP2 | 0.604 | 0.062 | (0.483,0.725) | 0 | 0 |
| CPNE2 | -0.596 | 0.011 | (-0.617,-0.575) | 0 | 0 |
| DRC7 | 0.668 | 0.001 | (0.665,0.671) | 0 | 0 |
| CCDC113 | 0.6 | 0.004 | (0.592,0.609) | 0 | 0 |
| CDH5 | -0.807 | 0.002 | (-0.811,-0.803) | 0 | 0 |
| AGRP | -1.221 | 0.164 | (-1.542,-0.899) | 0 | 0 |
| DPEP2 | -0.619 | 0.053 | (-0.722,-0.516) | 0 | 0 |
| DNAAF1 | 0.79 | 0.02 | (0.751,0.83) | 0 | 0 |
| FAM92B | 0.822 | 0.009 | (0.804,0.84) | 0 | 0 |
| INPP5K | -0.593 | 0.016 | (-0.624,-0.563) | 0 | 0 |
| SERPINF1 | 0.706 | 0.104 | (0.502,0.91) | 0 | 0 |
| ALOX15 | 0.732 | 0.004 | (0.724,0.74) | 0 | 0 |
| KIF1C | -0.587 | 0.008 | (-0.603,-0.571) | 0 | 0 |
| TEKT1 | 0.632 | 0.005 | (0.621,0.643) | 0 | 0 |
| TNFSF12 | -0.644 | 0.066 | (-0.774,-0.515) | 0 | 0 |
| DNAH2 | 0.654 | 0.002 | (0.651,0.657) | 0 | 0 |
| CFAP52 | 0.615 | 0.003 | (0.609,0.62) | 0 | 0 |
| DNAH9 | 0.655 | 0.004 | (0.647,0.663) | 0 | 0 |
| ALDH3A1 | 0.872 | 0.007 | (0.858,0.886) | 0 | 0 |
| SSH2 | -0.619 | 0.044 | (-0.706,-0.533) | 0 | 0 |
| SLC6A4 | -1.447 | 0.003 | (-1.453,-1.442) | 0 | 0 |
| CCL7 | 1.189 | 0.101 | (0.992,1.387) | 0 | 0 |
| GAS2L2 | 0.69 | 0.001 | (0.688,0.692) | 0 | 0 |
| DUSP14 | 0.682 | 0.007 | (0.669,0.695) | 0 | 0 |
| KRT15 | 1.408 | 0.031 | (1.348,1.468) | 0 | 0 |
| KRT17 | 1.367 | 0.098 | (1.175,1.559) | 0 | 0 |
| RAMP2 | -0.828 | 0.015 | (-0.856,-0.799) | 0 | 0 |
| LRRC46 | 0.605 | 0.008 | (0.589,0.621) | 0 | 0 |
| COL1A1 | 1.213 | 0.194 | (0.834,1.593) | 0 | 0 |
| TMEM100 | -1.172 | 0.008 | (-1.187,-1.157) | 0 | 0 |
| ANKFN1 | 0.613 | 0.001 | (0.611,0.614) | 0 | 0 |
| ACE | -0.663 | 0.036 | (-0.734,-0.592) | 0 | 0 |
| ICAM2 | -0.731 | 0.036 | (-0.802,-0.659) | 0 | 0 |
| DNAI2 | 0.641 | 0.003 | (0.635,0.647) | 0 | 0 |
| CD300LF | -0.716 | 0.095 | (-0.902,-0.531) | 0 | 0 |
| ITGB4 | 0.759 | 0.005 | (0.749,0.769) | 0 | 0 |
| ST6GALNAC1 | 0.687 | 0.009 | (0.669,0.705) | 0 | 0 |
| PTPRM | -0.649 | 0.008 | (-0.664,-0.634) | 0 | 0 |
| KCTD1 | 0.608 | 0.002 | (0.603,0.612) | 0 | 0 |
| CHST9 | 0.82 | 0.009 | (0.802,0.837) | 0 | 0 |
| SMAD7 | -0.666 | 0.02 | (-0.704,-0.627) | 0 | 0 |
| SIRPB1 | -0.784 | 0.077 | (-0.934,-0.634) | 0 | 0 |
| SPEF1 | 0.656 | 0.003 | (0.651,0.661) | 0 | 0 |
| ID1 | -0.641 | 0.036 | (-0.711,-0.571) | 0 | 0 |
| BPIFA1 | 0.972 | 0.067 | (0.839,1.104) | 0 | 0 |
| PTPRT | 0.686 | 0.001 | (0.684,0.688) | 0 | 0 |
| PREX1 | -0.59 | 0.026 | (-0.641,-0.539) | 0 | 0 |
| BCAS1 | 0.657 | 0.002 | (0.654,0.661) | 0 | 0 |
| C20orf85 | 0.816 | 0.052 | (0.713,0.918) | 0 | 0 |
| PLPP2 | 0.944 | 0.018 | (0.909,0.98) | 0 | 0 |
| MRPL54 | -0.598 | 0.092 | (-0.778,-0.419) | 0 | 0 |
| CAPS | 0.666 | 0.057 | (0.553,0.778) | 0 | 0 |
| PRAM1 | -0.597 | 0.049 | (-0.693,-0.501) | 0 | 0 |
| MUC16 | 1.034 | 0.01 | (1.014,1.055) | 0 | 0 |
| CDKN2D | -0.589 | 0.015 | (-0.618,-0.559) | 0 | 0 |
| GDF15 | 0.757 | 0.098 | (0.564,0.95) | 0 | 0 |
| LGALS7 | 1.096 | 0.014 | (1.069,1.124) | 0 | 0 |
| LGALS7B | 1.3 | 0.021 | (1.259,1.341) | 0 | 0 |
| PAK4 | -0.678 | 0.004 | (-0.686,-0.671) | 0 | 0 |
| CYP2F1 | 0.731 | 0.02 | (0.693,0.769) | 0 | 0 |
| KCNN4 | 0.882 | 0.032 | (0.819,0.945) | 0 | 0 |
| CCDC114 | 0.667 | 0.003 | (0.661,0.673) | 0 | 0 |
| SULT2B1 | -0.846 | 0.005 | (-0.855,-0.836) | 0 | 0 |
| RRAS | -0.594 | 0.012 | (-0.618,-0.569) | 0 | 0 |
| NKG7 | -0.862 | 0.128 | (-1.112,-0.611) | 0 | 0 |
| HAS1 | 0.636 | 0.085 | (0.469,0.803) | 0 | 0 |
| LILRA5 | -0.622 | 0.113 | (-0.843,-0.401) | 0 | 0 |
| LILRA2 | -0.689 | 0.058 | (-0.802,-0.576) | 0 | 0 |
| DNAAF3 | 0.65 | 0.003 | (0.645,0.656) | 0 | 0 |
| CLDN5 | -0.782 | 0.02 | (-0.821,-0.743) | 0 | 0 |
| LRRC74B | 0.608 | 0.001 | (0.606,0.609) | 0 | 0 |
| RSPH14 | 0.637 | 0.002 | (0.634,0.64) | 0 | 0 |
| C22orf15 | 0.642 | 0.004 | (0.635,0.65) | 0 | 0 |
| DERL3 | 1.301 | 0.039 | (1.225,1.378) | 0 | 0 |
| GGT5 | 0.621 | 0.004 | (0.614,0.629) | 0 | 0 |
| TIMP3 | -0.671 | 0.045 | (-0.76,-0.583) | 0 | 0 |
| CENPM | 0.599 | 0.013 | (0.573,0.624) | 0 | 0 |
| RIBC2 | 0.7 | 0.002 | (0.696,0.703) | 0 | 0 |
| CLIC6 | 0.765 | 0.006 | (0.753,0.778) | 0 | 0 |
| BACE2 | 0.72 | 0.012 | (0.697,0.743) | 0 | 0 |
| RSPH1 | 0.768 | 0.025 | (0.719,0.817) | 0 | 0 |
| IFNGR1 | -0.671 | 0.167 | (-0.998,-0.344) | 0 | 0.001 |
| TBXAS1 | -0.722 | 0.179 | (-1.072,-0.372) | 0 | 0.001 |
| SSR4 | 0.737 | 0.184 | (0.378,1.097) | 0 | 0.001 |
| LCN2 | 0.7 | 0.172 | (0.363,1.038) | 0 | 0.001 |
| MMP7 | 1.457 | 0.372 | (0.728,2.186) | 0 | 0.001 |
| FPR2 | -0.762 | 0.185 | (-1.124,-0.4) | 0 | 0.001 |
| TGFBI | 0.656 | 0.173 | (0.318,0.995) | 0 | 0.002 |
| ABCG1 | -0.655 | 0.169 | (-0.987,-0.323) | 0 | 0.002 |
| ACSL1 | -0.741 | 0.2 | (-1.132,-0.349) | 0 | 0.003 |
| CITED2 | -1.503 | 0.41 | (-2.306,-0.7) | 0 | 0.003 |
| COL1A2 | 0.838 | 0.228 | (0.392,1.285) | 0 | 0.003 |
| CLU | 0.659 | 0.176 | (0.315,1.003) | 0 | 0.003 |
| MMP9 | 1.143 | 0.308 | (0.54,1.747) | 0 | 0.003 |
| GMFG | -0.605 | 0.163 | (-0.925,-0.286) | 0 | 0.003 |
| CHIT1 | 1.486 | 0.411 | (0.681,2.291) | 0 | 0.004 |
| FCGR3A | -1.139 | 0.325 | (-1.775,-0.503) | 0 | 0.006 |
| MT1G | 0.608 | 0.174 | (0.266,0.95) | 0 | 0.007 |
| TFF3 | 0.635 | 0.185 | (0.272,0.997) | 0.001 | 0.008 |
| AGR2 | 0.74 | 0.216 | (0.316,1.164) | 0.001 | 0.009 |
| KRT19 | 0.654 | 0.192 | (0.277,1.03) | 0.001 | 0.009 |
| BLVRB | -0.791 | 0.233 | (-1.248,-0.334) | 0.001 | 0.01 |

**Table S4.** The estimation, standard error, 95% confidence interval (95% CI), p-value, adjusted p-value of $\beta_{1}$ from the detailed list of genes detected by Hybrid, informative method (436 genes).

| **Hybrid, informative** | | | | | |
| --- | --- | --- | --- | --- | --- |
| geneName | Estimate | Ste | CI | p_value | p_adjust |
| PRKCZ | -0.601 | 0.066 | (-0.731,-0.471) | 0 | 0 |
| TP73 | 0.659 | 0.083 | (0.496,0.823) | 0 | 0 |
| PLA2G2A | 1.597 | 0.269 | (1.071,2.124) | 0 | 0 |
| FCN3 | -1.29 | 0.156 | (-1.596,-0.985) | 0 | 0 |
| CD164L2 | 0.673 | 0.084 | (0.507,0.838) | 0 | 0 |
| CSF3R | -0.893 | 0.104 | (-1.097,-0.689) | 0 | 0 |
| MACF1 | -0.619 | 0.07 | (-0.756,-0.482) | 0 | 0 |
| FAM183A | 0.673 | 0.081 | (0.515,0.832) | 0 | 0 |
| CCDC17 | 0.587 | 0.073 | (0.443,0.73) | 0 | 0 |
| TSPAN1 | 0.861 | 0.097 | (0.671,1.051) | 0 | 0 |
| ERICH3 | 0.636 | 0.082 | (0.475,0.796) | 0 | 0 |
| GBP4 | -0.817 | 0.099 | (-1.012,-0.623) | 0 | 0 |
| S1PR1 | -0.7 | 0.077 | (-0.851,-0.549) | 0 | 0 |
| C1orf194 | 0.737 | 0.091 | (0.558,0.916) | 0 | 0 |
| PIFO | 0.655 | 0.078 | (0.501,0.808) | 0 | 0 |
| SPAG17 | 0.741 | 0.095 | (0.554,0.927) | 0 | 0 |
| FMO5 | -0.711 | 0.083 | (-0.873,-0.548) | 0 | 0 |
| S100A2 | 1.842 | 0.217 | (1.417,2.267) | 0 | 0 |
| SLC27A3 | -0.598 | 0.065 | (-0.726,-0.47) | 0 | 0 |
| KCNN3 | 1.035 | 0.125 | (0.789,1.28) | 0 | 0 |
| CRABP2 | 0.712 | 0.082 | (0.55,0.873) | 0 | 0 |
| ACKR1 | 1.019 | 0.12 | (0.784,1.253) | 0 | 0 |
| CFAP126 | 0.703 | 0.09 | (0.525,0.88) | 0 | 0 |
| FCGR3A | -1.14 | 0.262 | (-1.653,-0.628) | 0 | 0 |
| RCSD1 | -0.861 | 0.093 | (-1.043,-0.679) | 0 | 0 |
| KIAA0040 | -0.69 | 0.076 | (-0.839,-0.54) | 0 | 0 |
| APOBEC4 | 0.704 | 0.092 | (0.523,0.885) | 0 | 0 |
| RGS1 | 1.213 | 0.277 | (0.671,1.755) | 0 | 0 |
| CFH | 0.818 | 0.082 | (0.657,0.979) | 0 | 0 |
| CHIT1 | 1.486 | 0.311 | (0.876,2.096) | 0 | 0 |
| CTSE | 0.696 | 0.081 | (0.538,0.855) | 0 | 0 |
| PPP2R5A | -0.591 | 0.065 | (-0.718,-0.465) | 0 | 0 |
| C1orf198 | -0.859 | 0.09 | (-1.036,-0.682) | 0 | 0 |
| FAM89A | -0.763 | 0.105 | (-0.968,-0.557) | 0 | 0 |
| OPN3 | -0.647 | 0.096 | (-0.836,-0.458) | 0 | 0 |
| KLF11 | -0.655 | 0.057 | (-0.767,-0.544) | 0 | 0 |
| HPCAL1 | -1.095 | 0.113 | (-1.316,-0.874) | 0 | 0 |
| DRC1 | 0.59 | 0.077 | (0.439,0.741) | 0 | 0 |
| PRKCE | -0.755 | 0.079 | (-0.909,-0.6) | 0 | 0 |
| EPAS1 | -0.774 | 0.092 | (-0.955,-0.594) | 0 | 0 |
| SPTBN1 | -0.65 | 0.085 | (-0.816,-0.484) | 0 | 0 |
| GNLY | -0.828 | 0.159 | (-1.14,-0.517) | 0 | 0 |
| VWA3B | 0.611 | 0.079 | (0.456,0.765) | 0 | 0 |
| IL1RL1 | -0.82 | 0.106 | (-1.027,-0.613) | 0 | 0 |
| FHL2 | 1.24 | 0.128 | (0.989,1.492) | 0 | 0 |
| EPB41L5 | -1.094 | 0.123 | (-1.335,-0.854) | 0 | 0 |
| LIMS2 | -0.71 | 0.079 | (-0.865,-0.556) | 0 | 0 |
| CCDC74B | 0.586 | 0.073 | (0.442,0.73) | 0 | 0 |
| CCDC74A | 0.586 | 0.071 | (0.448,0.724) | 0 | 0 |
| MAP3K19 | 0.646 | 0.084 | (0.482,0.81) | 0 | 0 |
| CD302 | -1.221 | 0.106 | (-1.429,-1.013) | 0 | 0 |
| ZNF385B | -0.958 | 0.117 | (-1.187,-0.729) | 0 | 0 |
| CALCRL | -0.61 | 0.071 | (-0.748,-0.471) | 0 | 0 |
| TFPI | -0.649 | 0.074 | (-0.795,-0.503) | 0 | 0 |
| COL3A1 | 1.17 | 0.161 | (0.854,1.486) | 0 | 0 |
| SLC40A1 | -0.623 | 0.077 | (-0.773,-0.472) | 0 | 0 |
| ANKRD44 | -0.608 | 0.077 | (-0.758,-0.458) | 0 | 0 |
| KIAA2012 | 0.628 | 0.08 | (0.471,0.784) | 0 | 0 |
| BMPR2 | -0.615 | 0.067 | (-0.745,-0.484) | 0 | 0 |
| MDH1B | 0.602 | 0.075 | (0.454,0.75) | 0 | 0 |
| IGFBP2 | 0.884 | 0.126 | (0.637,1.13) | 0 | 0 |
| TNS1 | -0.623 | 0.07 | (-0.76,-0.486) | 0 | 0 |
| CFAP65 | 0.635 | 0.082 | (0.474,0.796) | 0 | 0 |
| TUBA4B | 0.73 | 0.094 | (0.545,0.915) | 0 | 0 |
| COL6A3 | 0.804 | 0.089 | (0.63,0.978) | 0 | 0 |
| RAMP1 | 0.619 | 0.071 | (0.479,0.758) | 0 | 0 |
| PPARG | -1.27 | 0.229 | (-1.718,-0.821) | 0 | 0 |
| FBLN2 | 0.86 | 0.098 | (0.668,1.052) | 0 | 0 |
| SH3BP5 | -0.654 | 0.104 | (-0.858,-0.449) | 0 | 0 |
| GPD1L | -0.614 | 0.064 | (-0.74,-0.489) | 0 | 0 |
| STAC | -0.611 | 0.079 | (-0.766,-0.457) | 0 | 0 |
| VIPR1 | -1.196 | 0.149 | (-1.489,-0.903) | 0 | 0 |
| ABHD5 | -0.974 | 0.161 | (-1.289,-0.658) | 0 | 0 |
| CCRL2 | -0.631 | 0.076 | (-0.781,-0.481) | 0 | 0 |
| CDHR4 | 0.696 | 0.09 | (0.519,0.873) | 0 | 0 |
| HYAL2 | -0.656 | 0.074 | (-0.802,-0.51) | 0 | 0 |
| ZMYND10 | 0.594 | 0.073 | (0.45,0.738) | 0 | 0 |
| CACNA2D2 | -0.91 | 0.11 | (-1.126,-0.694) | 0 | 0 |
| TNNC1 | -1.087 | 0.132 | (-1.346,-0.828) | 0 | 0 |
| DNAH12 | 0.671 | 0.085 | (0.505,0.838) | 0 | 0 |
| FAM107A | -0.739 | 0.092 | (-0.92,-0.558) | 0 | 0 |
| SNTN | 0.739 | 0.093 | (0.557,0.92) | 0 | 0 |
| TMEM45A | 0.869 | 0.089 | (0.694,1.045) | 0 | 0 |
| HHLA2 | 1.104 | 0.139 | (0.832,1.376) | 0 | 0 |
| CCDC80 | 0.957 | 0.149 | (0.666,1.248) | 0 | 0 |
| ARHGAP31 | -0.688 | 0.077 | (-0.839,-0.537) | 0 | 0 |
| CFAP100 | 0.624 | 0.08 | (0.466,0.782) | 0 | 0 |
| AMOTL2 | -0.643 | 0.07 | (-0.779,-0.507) | 0 | 0 |
| CLDN18 | -0.788 | 0.095 | (-0.974,-0.602) | 0 | 0 |
| PCOLCE2 | -0.627 | 0.113 | (-0.849,-0.405) | 0 | 0 |
| CP | 1 | 0.112 | (0.781,1.219) | 0 | 0 |
| ANKUB1 | 0.626 | 0.079 | (0.47,0.781) | 0 | 0 |
| MME | -1.221 | 0.149 | (-1.514,-0.928) | 0 | 0 |
| VEPH1 | -0.596 | 0.072 | (-0.738,-0.454) | 0 | 0 |
| RARRES1 | 1.018 | 0.105 | (0.812,1.225) | 0 | 0 |
| ZBBX | 0.703 | 0.092 | (0.523,0.882) | 0 | 0 |
| LAMP3 | -0.765 | 0.106 | (-0.972,-0.558) | 0 | 0 |
| ST6GAL1 | 0.65 | 0.062 | (0.528,0.771) | 0 | 0 |
| P3H2 | -0.871 | 0.103 | (-1.074,-0.669) | 0 | 0 |
| CLDN1 | 0.817 | 0.096 | (0.63,1.005) | 0 | 0 |
| MUC4 | 1.008 | 0.121 | (0.772,1.245) | 0 | 0 |
| CD38 | 0.746 | 0.092 | (0.566,0.926) | 0 | 0 |
| PROM1 | 0.955 | 0.122 | (0.716,1.193) | 0 | 0 |
| LIMCH1 | -0.816 | 0.09 | (-0.992,-0.64) | 0 | 0 |
| ATP8A1 | -0.703 | 0.077 | (-0.853,-0.553) | 0 | 0 |
| HOPX | -0.672 | 0.154 | (-0.974,-0.369) | 0 | 0 |
| CXCL6 | 0.767 | 0.096 | (0.578,0.955) | 0 | 0 |
| CDKL2 | -0.622 | 0.073 | (-0.765,-0.479) | 0 | 0 |
| ANXA3 | -0.993 | 0.117 | (-1.223,-0.764) | 0 | 0 |
| ADH1B | -0.682 | 0.103 | (-0.884,-0.48) | 0 | 0 |
| SLC39A8 | -0.945 | 0.122 | (-1.185,-0.705) | 0 | 0 |
| NPNT | -0.725 | 0.081 | (-0.883,-0.567) | 0 | 0 |
| CFI | 0.647 | 0.063 | (0.523,0.771) | 0 | 0 |
| HHIP | -0.81 | 0.1 | (-1.006,-0.615) | 0 | 0 |
| TTC29 | 0.602 | 0.078 | (0.449,0.756) | 0 | 0 |
| MSMO1 | -0.604 | 0.07 | (-0.74,-0.467) | 0 | 0 |
| HPGD | -1.174 | 0.185 | (-1.537,-0.812) | 0 | 0 |
| SPATA4 | 0.589 | 0.076 | (0.441,0.738) | 0 | 0 |
| WWC2 | -1.01 | 0.111 | (-1.229,-0.792) | 0 | 0 |
| ACSL1 | -0.741 | 0.166 | (-1.066,-0.417) | 0 | 0 |
| C5orf38 | -0.726 | 0.084 | (-0.89,-0.562) | 0 | 0 |
| C5orf49 | 0.66 | 0.08 | (0.502,0.818) | 0 | 0 |
| CAPSL | 0.753 | 0.097 | (0.563,0.944) | 0 | 0 |
| LIFR | -0.905 | 0.103 | (-1.106,-0.703) | 0 | 0 |
| C6 | 0.753 | 0.095 | (0.568,0.939) | 0 | 0 |
| HMGCS1 | -0.627 | 0.071 | (-0.766,-0.488) | 0 | 0 |
| CCNO | 0.904 | 0.116 | (0.677,1.131) | 0 | 0 |
| OCLN | -0.766 | 0.085 | (-0.934,-0.599) | 0 | 0 |
| FAM81B | 0.697 | 0.088 | (0.525,0.87) | 0 | 0 |
| PDLIM4 | 1.213 | 0.128 | (0.963,1.464) | 0 | 0 |
| CXCL14 | 1.382 | 0.164 | (1.06,1.703) | 0 | 0 |
| TGFBI | 0.656 | 0.144 | (0.373,0.939) | 0 | 0 |
| MZB1 | 1.377 | 0.186 | (1.012,1.743) | 0 | 0 |
| ADRB2 | -0.92 | 0.094 | (-1.105,-0.735) | 0 | 0 |
| GPX3 | -1.172 | 0.142 | (-1.451,-0.894) | 0 | 0 |
| PHACTR1 | -0.739 | 0.152 | (-1.036,-0.442) | 0 | 0 |
| MYLIP | -0.615 | 0.063 | (-0.739,-0.491) | 0 | 0 |
| STMND1 | 0.59 | 0.076 | (0.442,0.738) | 0 | 0 |
| RNF144B | -0.871 | 0.104 | (-1.075,-0.667) | 0 | 0 |
| SFTA2 | -0.83 | 0.147 | (-1.119,-0.541) | 0 | 0 |
| SLC44A4 | 0.63 | 0.074 | (0.485,0.774) | 0 | 0 |
| CFB | 0.775 | 0.085 | (0.609,0.941) | 0 | 0 |
| AGER | -1.459 | 0.175 | (-1.802,-1.117) | 0 | 0 |
| CCND3 | -0.628 | 0.08 | (-0.785,-0.471) | 0 | 0 |
| CLIC5 | -0.666 | 0.086 | (-0.835,-0.498) | 0 | 0 |
| PLA2G7 | 0.938 | 0.148 | (0.648,1.228) | 0 | 0 |
| ANKRD66 | 0.66 | 0.086 | (0.491,0.829) | 0 | 0 |
| ADGRF5 | -0.735 | 0.083 | (-0.897,-0.573) | 0 | 0 |
| GSTA1 | 0.994 | 0.127 | (0.745,1.243) | 0 | 0 |
| TPBG | 0.767 | 0.076 | (0.619,0.916) | 0 | 0 |
| CD24 | 0.683 | 0.069 | (0.548,0.818) | 0 | 0 |
| RSPH4A | 0.594 | 0.075 | (0.448,0.741) | 0 | 0 |
| IFNGR1 | -0.672 | 0.146 | (-0.957,-0.386) | 0 | 0 |
| ECT2L | 0.625 | 0.081 | (0.467,0.783) | 0 | 0 |
| CITED2 | -1.505 | 0.355 | (-2.201,-0.808) | 0 | 0 |
| PHACTR2 | -0.624 | 0.06 | (-0.741,-0.508) | 0 | 0 |
| STX11 | -1.06 | 0.117 | (-1.289,-0.831) | 0 | 0 |
| QKI | -0.667 | 0.076 | (-0.815,-0.519) | 0 | 0 |
| C6orf118 | 0.701 | 0.092 | (0.521,0.88) | 0 | 0 |
| ETV1 | -0.614 | 0.07 | (-0.751,-0.478) | 0 | 0 |
| AGR2 | 0.74 | 0.173 | (0.402,1.079) | 0 | 0 |
| RAPGEF5 | -0.788 | 0.089 | (-0.962,-0.614) | 0 | 0 |
| RAMP3 | -0.749 | 0.087 | (-0.921,-0.578) | 0 | 0 |
| ABCA13 | 0.676 | 0.086 | (0.506,0.845) | 0 | 0 |
| HIP1 | -0.632 | 0.07 | (-0.769,-0.494) | 0 | 0 |
| CCL24 | 1.511 | 0.349 | (0.827,2.196) | 0 | 0 |
| UPK3B | -0.765 | 0.1 | (-0.961,-0.569) | 0 | 0 |
| CD36 | -0.789 | 0.134 | (-1.051,-0.526) | 0 | 0 |
| COL1A2 | 0.839 | 0.196 | (0.454,1.223) | 0 | 0 |
| PILRA | -0.719 | 0.115 | (-0.945,-0.492) | 0 | 0 |
| MUC12 | 0.623 | 0.068 | (0.489,0.757) | 0 | 0 |
| EFCAB10 | 0.607 | 0.074 | (0.461,0.752) | 0 | 0 |
| DOCK4 | -0.634 | 0.074 | (-0.78,-0.489) | 0 | 0 |
| CAV2 | -0.592 | 0.066 | (-0.722,-0.462) | 0 | 0 |
| CAV1 | -0.935 | 0.113 | (-1.156,-0.715) | 0 | 0 |
| TSPAN12 | -0.873 | 0.098 | (-1.066,-0.681) | 0 | 0 |
| TBXAS1 | -0.722 | 0.149 | (-1.015,-0.429) | 0 | 0 |
| WWC3 | -0.602 | 0.06 | (-0.72,-0.484) | 0 | 0 |
| ARHGAP6 | -0.829 | 0.087 | (-0.999,-0.659) | 0 | 0 |
| TSPAN7 | -0.597 | 0.064 | (-0.723,-0.471) | 0 | 0 |
| MID1IP1 | -0.74 | 0.07 | (-0.878,-0.603) | 0 | 0 |
| MAOA | -0.745 | 0.083 | (-0.909,-0.582) | 0 | 0 |
| EFHC2 | 0.644 | 0.08 | (0.487,0.801) | 0 | 0 |
| PIM2 | 0.813 | 0.093 | (0.63,0.995) | 0 | 0 |
| MSN | -0.873 | 0.154 | (-1.175,-0.572) | 0 | 0 |
| PIH1D3 | 0.674 | 0.088 | (0.502,0.847) | 0 | 0 |
| AKAP14 | 0.647 | 0.085 | (0.481,0.813) | 0 | 0 |
| GPC3 | -0.634 | 0.073 | (-0.776,-0.492) | 0 | 0 |
| HMGB3 | 0.625 | 0.064 | (0.499,0.75) | 0 | 0 |
| SSR4 | 0.737 | 0.16 | (0.423,1.05) | 0 | 0 |
| DLC1 | -0.708 | 0.083 | (-0.872,-0.545) | 0 | 0 |
| DOK2 | -0.82 | 0.137 | (-1.088,-0.552) | 0 | 0 |
| PEBP4 | -0.879 | 0.107 | (-1.09,-0.669) | 0 | 0 |
| ADAM28 | 0.695 | 0.075 | (0.547,0.843) | 0 | 0 |
| BNIP3L | -0.687 | 0.129 | (-0.94,-0.433) | 0 | 0 |
| CLU | 0.659 | 0.155 | (0.356,0.963) | 0 | 0 |
| RAB11FIP1 | -0.692 | 0.099 | (-0.885,-0.498) | 0 | 0 |
| EFCAB1 | 0.629 | 0.079 | (0.474,0.785) | 0 | 0 |
| PPP1R42 | 0.632 | 0.081 | (0.473,0.792) | 0 | 0 |
| HEY1 | -0.741 | 0.089 | (-0.915,-0.568) | 0 | 0 |
| PAG1 | -0.765 | 0.085 | (-0.932,-0.599) | 0 | 0 |
| CA2 | -0.825 | 0.143 | (-1.105,-0.544) | 0 | 0 |
| GEM | 0.755 | 0.114 | (0.532,0.979) | 0 | 0 |
| COL14A1 | 1.311 | 0.152 | (1.014,1.608) | 0 | 0 |
| FBXO32 | 0.611 | 0.069 | (0.476,0.746) | 0 | 0 |
| DENND3 | -0.807 | 0.08 | (-0.964,-0.65) | 0 | 0 |
| LY6D | 1.369 | 0.172 | (1.033,1.705) | 0 | 0 |
| CDKN2A | 0.64 | 0.067 | (0.508,0.772) | 0 | 0 |
| C9orf24 | 0.729 | 0.087 | (0.558,0.9) | 0 | 0 |
| DNAI1 | 0.674 | 0.087 | (0.503,0.845) | 0 | 0 |
| UNC13B | -0.702 | 0.075 | (-0.849,-0.555) | 0 | 0 |
| PIP5K1B | -0.835 | 0.09 | (-1.012,-0.658) | 0 | 0 |
| C9orf135 | 0.748 | 0.098 | (0.556,0.939) | 0 | 0 |
| PRUNE2 | 0.599 | 0.07 | (0.463,0.736) | 0 | 0 |
| AGTPBP1 | -0.759 | 0.067 | (-0.89,-0.629) | 0 | 0 |
| GOLM1 | 0.765 | 0.078 | (0.613,0.917) | 0 | 0 |
| TNC | 0.788 | 0.089 | (0.613,0.963) | 0 | 0 |
| MORN5 | 0.627 | 0.08 | (0.471,0.783) | 0 | 0 |
| WDR38 | 0.703 | 0.091 | (0.524,0.882) | 0 | 0 |
| SCAI | -0.659 | 0.068 | (-0.793,-0.525) | 0 | 0 |
| CFAP157 | 0.623 | 0.078 | (0.47,0.776) | 0 | 0 |
| LCN2 | 0.701 | 0.148 | (0.411,0.99) | 0 | 0 |
| CFAP77 | 0.696 | 0.09 | (0.519,0.873) | 0 | 0 |
| EGFL7 | -0.697 | 0.073 | (-0.84,-0.554) | 0 | 0 |
| CLIC3 | -1.013 | 0.122 | (-1.251,-0.774) | 0 | 0 |
| LRRC26 | 0.801 | 0.099 | (0.607,0.996) | 0 | 0 |
| MUC5B | 1.259 | 0.156 | (0.953,1.565) | 0 | 0 |
| SYT8 | 0.801 | 0.091 | (0.622,0.98) | 0 | 0 |
| C11orf16 | 0.635 | 0.083 | (0.473,0.797) | 0 | 0 |
| SAA2 | 0.704 | 0.094 | (0.519,0.889) | 0 | 0 |
| MDK | 0.656 | 0.069 | (0.521,0.791) | 0 | 0 |
| MS4A8 | 0.673 | 0.084 | (0.508,0.839) | 0 | 0 |
| ASRGL1 | -0.654 | 0.072 | (-0.794,-0.513) | 0 | 0 |
| CTSW | -0.634 | 0.083 | (-0.796,-0.472) | 0 | 0 |
| DNAJB13 | 0.628 | 0.079 | (0.474,0.782) | 0 | 0 |
| ARRB1 | -0.825 | 0.078 | (-0.977,-0.672) | 0 | 0 |
| GAB2 | -0.605 | 0.062 | (-0.727,-0.484) | 0 | 0 |
| C11orf97 | 0.698 | 0.091 | (0.518,0.877) | 0 | 0 |
| MMP7 | 1.457 | 0.295 | (0.879,2.035) | 0 | 0 |
| C11orf88 | 0.606 | 0.077 | (0.456,0.757) | 0 | 0 |
| FXYD6 | -0.633 | 0.072 | (-0.774,-0.492) | 0 | 0 |
| TMPRSS4 | 1.467 | 0.183 | (1.109,1.825) | 0 | 0 |
| JAML | -0.591 | 0.098 | (-0.783,-0.398) | 0 | 0 |
| THY1 | 1.291 | 0.154 | (0.988,1.594) | 0 | 0 |
| UBASH3B | -0.8 | 0.089 | (-0.975,-0.625) | 0 | 0 |
| NRGN | -0.742 | 0.081 | (-0.901,-0.583) | 0 | 0 |
| VSIG2 | -0.623 | 0.071 | (-0.763,-0.483) | 0 | 0 |
| ESAM | -0.907 | 0.099 | (-1.1,-0.713) | 0 | 0 |
| ECHDC3 | -0.592 | 0.064 | (-0.718,-0.467) | 0 | 0 |
| NEBL | -0.778 | 0.088 | (-0.95,-0.606) | 0 | 0 |
| SPAG6 | 0.691 | 0.088 | (0.517,0.864) | 0 | 0 |
| ARMC3 | 0.692 | 0.089 | (0.518,0.866) | 0 | 0 |
| OTUD1 | -0.721 | 0.076 | (-0.87,-0.572) | 0 | 0 |
| ENKUR | 0.643 | 0.082 | (0.483,0.803) | 0 | 0 |
| CXCL12 | 0.618 | 0.076 | (0.47,0.767) | 0 | 0 |
| MSMB | 1.045 | 0.169 | (0.715,1.376) | 0 | 0 |
| FRMPD2 | 0.769 | 0.1 | (0.574,0.965) | 0 | 0 |
| STOX1 | 0.619 | 0.079 | (0.464,0.774) | 0 | 0 |
| PRF1 | -0.809 | 0.096 | (-0.998,-0.621) | 0 | 0 |
| SPOCK2 | -1.001 | 0.119 | (-1.235,-0.767) | 0 | 0 |
| SFTPD | -0.65 | 0.079 | (-0.805,-0.494) | 0 | 0 |
| DYDC2 | 0.59 | 0.077 | (0.44,0.741) | 0 | 0 |
| ADIRF | -0.801 | 0.114 | (-1.025,-0.577) | 0 | 0 |
| PAPSS2 | -0.844 | 0.166 | (-1.17,-0.518) | 0 | 0 |
| IFIT2 | -0.647 | 0.073 | (-0.79,-0.503) | 0 | 0 |
| IFIT3 | -0.737 | 0.11 | (-0.954,-0.521) | 0 | 0 |
| FFAR4 | -0.741 | 0.085 | (-0.909,-0.574) | 0 | 0 |
| CRTAC1 | -1.09 | 0.131 | (-1.346,-0.835) | 0 | 0 |
| CFAP43 | 0.622 | 0.079 | (0.466,0.777) | 0 | 0 |
| PLEKHS1 | 1.042 | 0.133 | (0.782,1.302) | 0 | 0 |
| PLEKHA1 | -0.59 | 0.063 | (-0.713,-0.466) | 0 | 0 |
| FANK1 | 0.634 | 0.077 | (0.483,0.785) | 0 | 0 |
| CFAP46 | 0.647 | 0.083 | (0.484,0.81) | 0 | 0 |
| NINJ2 | -0.755 | 0.081 | (-0.913,-0.597) | 0 | 0 |
| CCND2 | 0.744 | 0.078 | (0.592,0.896) | 0 | 0 |
| CLEC4E | -0.741 | 0.095 | (-0.927,-0.555) | 0 | 0 |
| KLRB1 | -0.621 | 0.07 | (-0.758,-0.484) | 0 | 0 |
| CLEC12A | -0.781 | 0.1 | (-0.976,-0.586) | 0 | 0 |
| CLEC7A | -0.804 | 0.131 | (-1.06,-0.547) | 0 | 0 |
| GABARAPL1 | -0.647 | 0.128 | (-0.898,-0.396) | 0 | 0 |
| KLRD1 | -0.829 | 0.097 | (-1.019,-0.639) | 0 | 0 |
| PPFIBP1 | -0.684 | 0.078 | (-0.837,-0.531) | 0 | 0 |
| LRRK2 | -0.658 | 0.074 | (-0.804,-0.513) | 0 | 0 |
| AMIGO2 | -0.611 | 0.064 | (-0.737,-0.485) | 0 | 0 |
| FKBP11 | 0.731 | 0.075 | (0.585,0.878) | 0 | 0 |
| AQP5 | 0.992 | 0.117 | (0.762,1.221) | 0 | 0 |
| ACVRL1 | -0.954 | 0.103 | (-1.155,-0.752) | 0 | 0 |
| GRASP | -0.643 | 0.078 | (-0.797,-0.49) | 0 | 0 |
| KRT5 | 1.467 | 0.181 | (1.111,1.822) | 0 | 0 |
| RBMS2 | -0.666 | 0.069 | (-0.802,-0.531) | 0 | 0 |
| HSD17B6 | -1.127 | 0.132 | (-1.385,-0.869) | 0 | 0 |
| PTPRB | -0.936 | 0.106 | (-1.144,-0.728) | 0 | 0 |
| TSPAN19 | 0.636 | 0.08 | (0.479,0.794) | 0 | 0 |
| LRRIQ1 | 0.598 | 0.075 | (0.451,0.745) | 0 | 0 |
| CHPT1 | -0.678 | 0.07 | (-0.814,-0.541) | 0 | 0 |
| IGF1 | 1.088 | 0.126 | (0.841,1.335) | 0 | 0 |
| SELPLG | -0.589 | 0.086 | (-0.758,-0.419) | 0 | 0 |
| TRPV4 | 0.663 | 0.081 | (0.504,0.823) | 0 | 0 |
| CFAP73 | 0.656 | 0.084 | (0.491,0.82) | 0 | 0 |
| CCDC60 | 0.668 | 0.084 | (0.502,0.833) | 0 | 0 |
| PLA2G1B | -0.938 | 0.116 | (-1.165,-0.71) | 0 | 0 |
| OASL | -0.858 | 0.104 | (-1.061,-0.654) | 0 | 0 |
| LRRC43 | 0.616 | 0.077 | (0.465,0.767) | 0 | 0 |
| FRY | -0.752 | 0.078 | (-0.906,-0.598) | 0 | 0 |
| CCNA1 | 0.66 | 0.084 | (0.495,0.825) | 0 | 0 |
| POSTN | 1.317 | 0.153 | (1.018,1.617) | 0 | 0 |
| STOML3 | 0.704 | 0.092 | (0.523,0.885) | 0 | 0 |
| FAM216B | 0.691 | 0.089 | (0.516,0.866) | 0 | 0 |
| SPRYD7 | -0.779 | 0.089 | (-0.953,-0.605) | 0 | 0 |
| LMO7 | -0.708 | 0.088 | (-0.88,-0.536) | 0 | 0 |
| EDNRB | -1.022 | 0.113 | (-1.243,-0.8) | 0 | 0 |
| DOCK9 | -0.63 | 0.068 | (-0.763,-0.497) | 0 | 0 |
| CLEC14A | -0.729 | 0.082 | (-0.889,-0.569) | 0 | 0 |
| SIX1 | 0.907 | 0.113 | (0.685,1.128) | 0 | 0 |
| JDP2 | -0.664 | 0.06 | (-0.782,-0.545) | 0 | 0 |
| TJP1 | -0.665 | 0.071 | (-0.804,-0.527) | 0 | 0 |
| C15orf48 | 1.813 | 0.313 | (1.201,2.426) | 0 | 0 |
| GLDN | -1.243 | 0.167 | (-1.569,-0.916) | 0 | 0 |
| CCDC33 | 0.732 | 0.095 | (0.545,0.919) | 0 | 0 |
| CFAP161 | -0.615 | 0.077 | (-0.765,-0.464) | 0 | 0 |
| ALDH1A3 | 1.143 | 0.127 | (0.894,1.393) | 0 | 0 |
| HBA2 | -0.737 | 0.091 | (-0.915,-0.559) | 0 | 0 |
| CCDC78 | 0.649 | 0.081 | (0.49,0.808) | 0 | 0 |
| SLC9A3R2 | -0.906 | 0.101 | (-1.103,-0.709) | 0 | 0 |
| ABCA3 | -0.675 | 0.082 | (-0.836,-0.514) | 0 | 0 |
| EMP2 | -0.674 | 0.085 | (-0.841,-0.507) | 0 | 0 |
| DNAH3 | 0.624 | 0.079 | (0.469,0.78) | 0 | 0 |
| SULT1A1 | -0.75 | 0.071 | (-0.888,-0.611) | 0 | 0 |
| SPN | -0.693 | 0.084 | (-0.858,-0.528) | 0 | 0 |
| MMP2 | 0.604 | 0.076 | (0.455,0.753) | 0 | 0 |
| MT1G | 0.608 | 0.135 | (0.342,0.873) | 0 | 0 |
| CPNE2 | -0.596 | 0.06 | (-0.714,-0.478) | 0 | 0 |
| DRC7 | 0.668 | 0.086 | (0.5,0.837) | 0 | 0 |
| CCDC113 | 0.601 | 0.074 | (0.456,0.745) | 0 | 0 |
| CDH5 | -0.807 | 0.092 | (-0.988,-0.627) | 0 | 0 |
| AGRP | -1.222 | 0.158 | (-1.53,-0.913) | 0 | 0 |
| DPEP2 | -0.619 | 0.068 | (-0.753,-0.486) | 0 | 0 |
| DNAAF1 | 0.791 | 0.102 | (0.591,0.991) | 0 | 0 |
| FAM92B | 0.823 | 0.105 | (0.616,1.029) | 0 | 0 |
| INPP5K | -0.593 | 0.056 | (-0.704,-0.483) | 0 | 0 |
| SERPINF1 | 0.706 | 0.107 | (0.497,0.916) | 0 | 0 |
| ALOX15 | 0.732 | 0.095 | (0.546,0.918) | 0 | 0 |
| KIF1C | -0.587 | 0.061 | (-0.708,-0.467) | 0 | 0 |
| TEKT1 | 0.632 | 0.081 | (0.473,0.791) | 0 | 0 |
| TNFSF12 | -0.645 | 0.071 | (-0.784,-0.506) | 0 | 0 |
| DNAH2 | 0.654 | 0.082 | (0.493,0.815) | 0 | 0 |
| CFAP52 | 0.615 | 0.079 | (0.46,0.77) | 0 | 0 |
| DNAH9 | 0.656 | 0.083 | (0.493,0.818) | 0 | 0 |
| ALDH3A1 | 0.872 | 0.106 | (0.665,1.08) | 0 | 0 |
| SSH2 | -0.619 | 0.048 | (-0.714,-0.525) | 0 | 0 |
| SLC6A4 | -1.448 | 0.191 | (-1.821,-1.074) | 0 | 0 |
| CCL7 | 1.19 | 0.131 | (0.933,1.446) | 0 | 0 |
| GAS2L2 | 0.691 | 0.088 | (0.517,0.864) | 0 | 0 |
| DUSP14 | 0.682 | 0.079 | (0.528,0.836) | 0 | 0 |
| KRT15 | 1.408 | 0.162 | (1.091,1.726) | 0 | 0 |
| KRT19 | 0.654 | 0.158 | (0.345,0.963) | 0 | 0 |
| KRT17 | 1.368 | 0.144 | (1.085,1.65) | 0 | 0 |
| RAMP2 | -0.828 | 0.094 | (-1.012,-0.643) | 0 | 0 |
| LRRC46 | 0.605 | 0.076 | (0.456,0.754) | 0 | 0 |
| COL1A1 | 1.214 | 0.196 | (0.829,1.598) | 0 | 0 |
| TMEM100 | -1.172 | 0.147 | (-1.461,-0.883) | 0 | 0 |
| ANKFN1 | 0.613 | 0.077 | (0.463,0.763) | 0 | 0 |
| ACE | -0.663 | 0.087 | (-0.834,-0.493) | 0 | 0 |
| ICAM2 | -0.731 | 0.062 | (-0.853,-0.609) | 0 | 0 |
| DNAI2 | 0.641 | 0.083 | (0.479,0.803) | 0 | 0 |
| CD300LF | -0.717 | 0.09 | (-0.893,-0.54) | 0 | 0 |
| ITGB4 | 0.759 | 0.083 | (0.598,0.921) | 0 | 0 |
| ST6GALNAC1 | 0.687 | 0.079 | (0.533,0.841) | 0 | 0 |
| PTPRM | -0.649 | 0.063 | (-0.772,-0.526) | 0 | 0 |
| KCTD1 | 0.608 | 0.066 | (0.478,0.738) | 0 | 0 |
| CHST9 | 0.82 | 0.105 | (0.614,1.026) | 0 | 0 |
| SMAD7 | -0.666 | 0.068 | (-0.8,-0.532) | 0 | 0 |
| SIRPB1 | -0.784 | 0.101 | (-0.983,-0.586) | 0 | 0 |
| SPEF1 | 0.656 | 0.084 | (0.49,0.822) | 0 | 0 |
| ID1 | -0.641 | 0.089 | (-0.816,-0.466) | 0 | 0 |
| BPIFA1 | 0.972 | 0.124 | (0.729,1.215) | 0 | 0 |
| PTPRT | 0.686 | 0.088 | (0.514,0.859) | 0 | 0 |
| MMP9 | 1.143 | 0.236 | (0.679,1.607) | 0 | 0 |
| PREX1 | -0.59 | 0.057 | (-0.702,-0.479) | 0 | 0 |
| BCAS1 | 0.658 | 0.081 | (0.5,0.816) | 0 | 0 |
| C20orf85 | 0.816 | 0.099 | (0.622,1.01) | 0 | 0 |
| PLPP2 | 0.945 | 0.107 | (0.735,1.154) | 0 | 0 |
| MRPL54 | -0.599 | 0.097 | (-0.789,-0.408) | 0 | 0 |
| CAPS | 0.666 | 0.087 | (0.496,0.836) | 0 | 0 |
| PRAM1 | -0.597 | 0.057 | (-0.71,-0.485) | 0 | 0 |
| MUC16 | 1.035 | 0.132 | (0.775,1.294) | 0 | 0 |
| CDKN2D | -0.589 | 0.054 | (-0.695,-0.482) | 0 | 0 |
| GDF15 | 0.757 | 0.12 | (0.521,0.993) | 0 | 0 |
| LGALS7 | 1.097 | 0.14 | (0.823,1.371) | 0 | 0 |
| LGALS7B | 1.3 | 0.165 | (0.978,1.623) | 0 | 0 |
| PAK4 | -0.678 | 0.072 | (-0.82,-0.537) | 0 | 0 |
| GMFG | -0.606 | 0.146 | (-0.891,-0.321) | 0 | 0 |
| CYP2F1 | 0.732 | 0.092 | (0.552,0.911) | 0 | 0 |
| KCNN4 | 0.882 | 0.083 | (0.719,1.046) | 0 | 0 |
| CCDC114 | 0.667 | 0.085 | (0.5,0.835) | 0 | 0 |
| SULT2B1 | -0.846 | 0.1 | (-1.043,-0.649) | 0 | 0 |
| RRAS | -0.594 | 0.067 | (-0.725,-0.463) | 0 | 0 |
| NKG7 | -0.862 | 0.146 | (-1.149,-0.575) | 0 | 0 |
| HAS1 | 0.636 | 0.093 | (0.453,0.819) | 0 | 0 |
| FPR2 | -0.763 | 0.144 | (-1.045,-0.48) | 0 | 0 |
| LILRA5 | -0.622 | 0.105 | (-0.828,-0.416) | 0 | 0 |
| LILRA2 | -0.689 | 0.077 | (-0.84,-0.538) | 0 | 0 |
| DNAAF3 | 0.651 | 0.083 | (0.488,0.813) | 0 | 0 |
| CLDN5 | -0.782 | 0.089 | (-0.958,-0.607) | 0 | 0 |
| LRRC74B | 0.608 | 0.079 | (0.454,0.762) | 0 | 0 |
| RSPH14 | 0.637 | 0.082 | (0.477,0.797) | 0 | 0 |
| C22orf15 | 0.643 | 0.082 | (0.483,0.802) | 0 | 0 |
| DERL3 | 1.302 | 0.153 | (1.002,1.601) | 0 | 0 |
| GGT5 | 0.621 | 0.068 | (0.489,0.754) | 0 | 0 |
| TIMP3 | -0.672 | 0.092 | (-0.852,-0.492) | 0 | 0 |
| CENPM | 0.599 | 0.069 | (0.464,0.734) | 0 | 0 |
| RIBC2 | 0.7 | 0.088 | (0.527,0.872) | 0 | 0 |
| CLIC6 | 0.766 | 0.087 | (0.595,0.936) | 0 | 0 |
| BACE2 | 0.72 | 0.071 | (0.58,0.86) | 0 | 0 |
| ABCG1 | -0.656 | 0.142 | (-0.933,-0.378) | 0 | 0 |
| TFF3 | 0.635 | 0.15 | (0.341,0.929) | 0 | 0 |
| RSPH1 | 0.769 | 0.096 | (0.581,0.956) | 0 | 0 |
| CTSK | 0.706 | 0.173 | (0.368,1.045) | 0 | 0.001 |
| GYPC | -0.684 | 0.174 | (-1.025,-0.343) | 0 | 0.001 |
| GCA | -0.658 | 0.163 | (-0.978,-0.338) | 0 | 0.001 |
| CSTA | -0.749 | 0.191 | (-1.124,-0.374) | 0 | 0.001 |
| TREM1 | -1.123 | 0.279 | (-1.67,-0.577) | 0 | 0.001 |
| RHOG | -0.66 | 0.16 | (-0.974,-0.346) | 0 | 0.001 |
| BLVRB | -0.792 | 0.202 | (-1.187,-0.397) | 0 | 0.001 |
| CARD16 | -0.65 | 0.172 | (-0.986,-0.314) | 0 | 0.002 |
| ALOX5 | -0.894 | 0.237 | (-1.358,-0.43) | 0 | 0.002 |
| SLC7A7 | -0.995 | 0.261 | (-1.507,-0.482) | 0 | 0.002 |
| FPR1 | -0.59 | 0.155 | (-0.894,-0.287) | 0 | 0.002 |
| VSIG4 | -2.132 | 0.591 | (-3.289,-0.974) | 0 | 0.004 |
| PDK4 | -1.29 | 0.367 | (-2.01,-0.571) | 0 | 0.006 |
| IFI27 | -0.852 | 0.243 | (-1.328,-0.375) | 0 | 0.006 |
| NCF2 | -0.788 | 0.228 | (-1.235,-0.342) | 0.001 | 0.007 |
| SNX10 | -1.021 | 0.293 | (-1.596,-0.447) | 0 | 0.007 |
| MT1X | 0.923 | 0.269 | (0.396,1.45) | 0.001 | 0.008 |
| C1orf162 | -1.323 | 0.387 | (-2.083,-0.564) | 0.001 | 0.009 |
| BPIFB1 | 2.454 | 0.727 | (1.029,3.878) | 0.001 | 0.01 |

**Table S5.** A detailed list of pathways detected by Bayesian, informative method (36 pathways).

| **Bayesian, informative** | | | | | | | |
| --- | --- | --- | --- | --- | --- | --- | --- |
| Pathways | r | R | n | N | Zscore | pvalue | qvalue |
| Role of TGF-beta 1 in fibrosis development after myocardial infarction | 10 | 219 | 38 | 12814 | 11.72026 | 5.39E-10 | 8.23E-07 |
| IL-1 beta- and Endothelin-1-induced fibroblast/ myofibroblast migration and extracellular matrix production in asthmatic airways | 8 | 219 | 40 | 12814 | 8.93905 | 3.08E-07 | 0.000173 |
| Cell adhesion_ECM remodeling | 9 | 219 | 55 | 12814 | 8.403013 | 3.39E-07 | 0.000173 |
| TGF-beta-induced fibroblast/ myofibroblast migration and extracellular matrix production in asthmatic airways | 9 | 219 | 60 | 12814 | 7.961536 | 7.33E-07 | 0.00028 |
| Th2 cytokine- and TNF-alpha-induced profibrotic response in asthmatic airway fibroblasts/ myofibroblasts | 8 | 219 | 52 | 12814 | 7.623879 | 2.52E-06 | 0.000771 |
| Immune response_CCL2 signaling | 8 | 219 | 54 | 12814 | 7.445987 | 3.39E-06 | 0.000863 |
| TGF-beta 1-mediated induction of EMT in normal and asthmatic airway epithelium | 7 | 219 | 44 | 12814 | 7.279623 | 8.66E-06 | 0.00189 |
| Development_Inhibition of angiogenesis and regulation of endothelial cell function by PEDF | 8 | 219 | 64 | 12814 | 6.677022 | 1.25E-05 | 0.002377 |
| Immune response_IL-4-responsive genes in type 2 immunity | 8 | 219 | 70 | 12814 | 6.291138 | 2.43E-05 | 0.003892 |
| Role of fibroblasts in the sensitization phase of allergic contact dermatitis | 5 | 219 | 22 | 12814 | 7.61249 | 2.89E-05 | 0.003892 |
| Transition of Monoclonal gammopathy of undetermined significance to active multiple myeloma (schema) | 5 | 219 | 22 | 12814 | 7.61249 | 2.89E-05 | 0.003892 |
| Immune response_Alternative complement pathway | 7 | 219 | 53 | 12814 | 6.471798 | 3.06E-05 | 0.003892 |
| Cell cycle_Regulation of G1/S transition (part 1) | 6 | 219 | 38 | 12814 | 6.70654 | 4.07E-05 | 0.004778 |
| Cell adhesion_Endothelial cell contacts by junctional mechanisms | 5 | 219 | 26 | 12814 | 6.900024 | 6.84E-05 | 0.006558 |
| Hypothetical role of microRNAs in fibrosis development after myocardial infarction | 5 | 219 | 26 | 12814 | 6.900024 | 6.84E-05 | 0.006558 |
| Role of stellate cells in progression of pancreatic cancer | 7 | 219 | 60 | 12814 | 5.964802 | 6.93E-05 | 0.006558 |
| Stromal-epithelial interaction in Prostate Cancer | 6 | 219 | 42 | 12814 | 6.298672 | 7.3E-05 | 0.006558 |
| Tumor infiltrating cells: selected markers and effector molecules | 11 | 219 | 158 | 12814 | 5.125936 | 8.44E-05 | 0.006895 |
| Immune response_Regulatory role of C1q in platelet activation | 4 | 219 | 15 | 12814 | 7.461899 | 9.77E-05 | 0.006895 |
| Cell cycle_Regulation of G1/S transition (part 2) | 5 | 219 | 28 | 12814 | 6.599658 | 9.93E-05 | 0.006895 |
| Tumor-stroma interactions in pancreatic cancer | 5 | 219 | 28 | 12814 | 6.599658 | 9.93E-05 | 0.006895 |
| Regulation of Beta-catenin activity in melanoma | 5 | 219 | 28 | 12814 | 6.599658 | 9.93E-05 | 0.006895 |
| Development_Regulation of epithelial-to-mesenchymal transition (EMT) | 7 | 219 | 64 | 12814 | 5.710206 | 0.000105 | 0.006981 |
| Regulation of IGF family activity in colorectal cancer | 5 | 219 | 30 | 12814 | 6.32817 | 0.00014 | 0.008913 |
| Alternative complement cascade disruption in age-related macular degeneration | 5 | 219 | 31 | 12814 | 6.201798 | 0.000165 | 0.010062 |
| Cigarette smoke-induced Oxidative stress in progression of Lung fibrosis | 10 | 219 | 145 | 12814 | 4.846839 | 0.000192 | 0.011247 |
| Role of metalloproteases and heparanase in progression of pancreatic cancer | 5 | 219 | 33 | 12814 | 5.965432 | 0.000224 | 0.012657 |
| Development_Role of G-CSF in hematopoietic stem cell mobilization | 4 | 219 | 21 | 12814 | 6.135151 | 0.000395 | 0.021559 |
| Cell adhesion_Gap junctions | 4 | 219 | 22 | 12814 | 5.966192 | 0.000477 | 0.025103 |
| Role of alpha-V/ beta-6 integrin in colorectal cancer | 4 | 219 | 23 | 12814 | 5.80776 | 0.000569 | 0.028984 |
| Hedgehog signaling in breast cancer | 4 | 219 | 24 | 12814 | 5.658759 | 0.000674 | 0.033211 |
| Resolution of inflammation in healing myocardial infarction | 4 | 219 | 25 | 12814 | 5.518248 | 0.000792 | 0.036648 |
| Defective macrophage-mediated bacterial phagocytosis in COPD | 4 | 219 | 25 | 12814 | 5.518248 | 0.000792 | 0.036648 |
| Cell adhesion_Tight junctions | 5 | 219 | 44 | 12814 | 4.949402 | 0.000879 | 0.039491 |
| Inhibition of TGF-beta 1 signaling in early colorectal cancer | 4 | 219 | 27 | 12814 | 5.259547 | 0.00107 | 0.046673 |
| Chemotaxis_CXCR3-A signaling | 6 | 219 | 69 | 12814 | 4.489609 | 0.001138 | 0.048263 |

*Threshold: qvalue < 0.05; r: intersection of intology term with experiment list; R: size of experiment list; n: size of ontology term; N: size of background list; zscore: z-score of enrichment; pvalue: hypergeometric test enrichment p-value; qvalue: FDR-adjusted pvalue.

**Table S6.** A detailed list of pathways detected by Hybrid, informative method (38 pathways).

| **Hybrid, informative** | | | | | | | |
| --- | --- | --- | --- | --- | --- | --- | --- |
|  | r | R | n | N | Zscore | pvalue | qvalue |
| Role of TGF-beta 1 in fibrosis development after myocardial infarction | 10 | 236 | 38 | 12814 | 11.23695 | 1.12E-09 | 1.71E-06 |
| IL-1 beta- and Endothelin-1-induced fibroblast/ myofibroblast migration and extracellular matrix production in asthmatic airways | 8 | 236 | 40 | 12814 | 8.554393 | 5.44E-07 | 0.000324 |
| Cell adhesion_ECM remodeling | 9 | 236 | 55 | 12814 | 8.026844 | 6.37E-07 | 0.000324 |
| TGF-beta-induced fibroblast/ myofibroblast migration and extracellular matrix production in asthmatic airways | 9 | 236 | 60 | 12814 | 7.598001 | 1.37E-06 | 0.000522 |
| Development_Inhibition of angiogenesis and regulation of endothelial cell function by PEDF | 9 | 236 | 64 | 12814 | 7.289229 | 2.39E-06 | 0.000729 |
| Th2 cytokine- and TNF-alpha-induced profibrotic response in asthmatic airway fibroblasts/ myofibroblasts | 8 | 236 | 52 | 12814 | 7.277826 | 4.4E-06 | 0.000978 |
| Immune response_Alternative complement pathway | 8 | 236 | 53 | 12814 | 7.190269 | 5.1E-06 | 0.000978 |
| Immune response_IL-4-responsive genes in type 2 immunity | 9 | 236 | 70 | 12814 | 6.872979 | 5.12E-06 | 0.000978 |
| Immune response_CCL2 signaling | 8 | 236 | 54 | 12814 | 7.104981 | 5.89E-06 | 0.001 |
| TGF-beta 1-mediated induction of EMT in normal and asthmatic airway epithelium | 7 | 236 | 44 | 12814 | 6.951711 | 1.41E-05 | 0.002153 |
| Development_Role of G-CSF in hematopoietic stem cell mobilization | 5 | 236 | 21 | 12814 | 7.493041 | 3.25E-05 | 0.004508 |
| Tumor infiltrating cells: selected markers and effector molecules | 12 | 236 | 158 | 12814 | 5.411756 | 3.54E-05 | 0.004509 |
| Role of fibroblasts in the sensitization phase of allergic contact dermatitis | 5 | 236 | 22 | 12814 | 7.291823 | 4.14E-05 | 0.004516 |
| Transition of Monoclonal gammopathy of undetermined significance to active multiple myeloma (schema) | 5 | 236 | 22 | 12814 | 7.291823 | 4.14E-05 | 0.004516 |
| Cell cycle_Regulation of G1/S transition (part 1) | 6 | 236 | 38 | 12814 | 6.403928 | 6.17E-05 | 0.006283 |
| Cell adhesion_Endothelial cell contacts by junctional mechanisms | 5 | 236 | 26 | 12814 | 6.600991 | 9.74E-05 | 0.008747 |
| Hypothetical role of microRNAs in fibrosis development after myocardial infarction | 5 | 236 | 26 | 12814 | 6.600991 | 9.74E-05 | 0.008747 |
| Stromal-epithelial interaction in Prostate Cancer | 6 | 236 | 42 | 12814 | 6.007625 | 0.00011 | 0.008894 |
| Role of stellate cells in progression of pancreatic cancer | 7 | 236 | 60 | 12814 | 5.673228 | 0.000111 | 0.008894 |
| Immune response_Regulatory role of C1q in platelet activation | 4 | 236 | 15 | 12814 | 7.154742 | 0.000131 | 0.009374 |
| Cell cycle_Regulation of G1/S transition (part 2) | 5 | 236 | 28 | 12814 | 6.309544 | 0.000141 | 0.009374 |
| Tumor-stroma interactions in pancreatic cancer | 5 | 236 | 28 | 12814 | 6.309544 | 0.000141 | 0.009374 |
| Regulation of Beta-catenin activity in melanoma | 5 | 236 | 28 | 12814 | 6.309544 | 0.000141 | 0.009374 |
| Development_Regulation of epithelial-to-mesenchymal transition (EMT) | 7 | 236 | 64 | 12814 | 5.425283 | 0.000167 | 0.010641 |
| Regulation of IGF family activity in colorectal cancer | 5 | 236 | 30 | 12814 | 6.046002 | 0.000199 | 0.012135 |
| Alternative complement cascade disruption in age-related macular degeneration | 5 | 236 | 31 | 12814 | 5.923288 | 0.000233 | 0.013705 |
| Development_Role of proteases in hematopoietic stem cell mobilization | 4 | 236 | 18 | 12814 | 6.4352 | 0.00028 | 0.015842 |
| Role of metalloproteases and heparanase in progression of pancreatic cancer | 5 | 236 | 33 | 12814 | 5.693689 | 0.000316 | 0.017251 |
| Cigarette smoke-induced Oxidative stress in progression of Lung fibrosis | 10 | 236 | 145 | 12814 | 4.552674 | 0.000349 | 0.018374 |
| Basophil migration in asthma | 6 | 236 | 55 | 12814 | 5.011895 | 0.000499 | 0.025416 |
| Cell adhesion_Gap junctions | 4 | 236 | 22 | 12814 | 5.704856 | 0.000632 | 0.030813 |
| Cytoskeleton remodeling_Regulation of actin cytoskeleton organization by the kinase effectors of Rho GTPases | 6 | 236 | 58 | 12814 | 4.827052 | 0.000666 | 0.030813 |
| Role of integrins in eosinophil degranulation in asthma | 6 | 236 | 58 | 12814 | 4.827052 | 0.000666 | 0.030813 |
| Role of alpha-V/ beta-6 integrin in colorectal cancer | 4 | 236 | 23 | 12814 | 5.551091 | 0.000754 | 0.033865 |
| Hedgehog signaling in breast cancer | 4 | 236 | 24 | 12814 | 5.40644 | 0.000892 | 0.038911 |
| Resolution of inflammation in healing myocardial infarction | 4 | 236 | 25 | 12814 | 5.269993 | 0.001047 | 0.043191 |
| Defective macrophage-mediated bacterial phagocytosis in COPD | 4 | 236 | 25 | 12814 | 5.269993 | 0.001047 | 0.043191 |
| Cell adhesion_Tight junctions | 5 | 236 | 44 | 12814 | 4.705469 | 0.001228 | 0.049348 |

*Threshold: qvalue < 0.05; r: intersection of intology term with experiment list; R: size of experiment list; n: size of ontology term; N: size of background list; zscore: z-score of enrichment; pvalue: hypergeometric test enrichment p-value; qvalue: FDR-adjusted pvalue.
